# Resource Partitioning among “Ancillary” Pelagic Fishes (*Scomber* spp., *Trachurus* spp.) in the Adriatic Sea

**DOI:** 10.3390/biology12020272

**Published:** 2023-02-08

**Authors:** Zaira Da Ros, Emanuela Fanelli, Sacha Cassatella, Ilaria Biagiotti, Giovanni Canduci, Samuele Menicucci, Andrea De Felice, Sara Malavolti, Iole Leonori

**Affiliations:** 1 Department of Life and Environmental Sciences, Polytechnic University of Marche, 60131 Ancona, Italy; 2 IRBIM-Institute of Marine Biological Resources and Biotechnologies, CNR-National Research Council, Largo Fiera della Pesca, 1, 60125 Ancona, Italy

**Keywords:** pelagic food web, stable isotopes, stomach contents, Atlantic mackerel, Atlantic chub mackerel, Mediterranean horse mackerel, Atlantic horse mackerel, MEDIAS, Mediterranean Sea

## Abstract

**Simple Summary:**

In the Adriatic Sea, there is little knowledge concerning the role of medium-sized pelagic fish species such as *Scomber* spp. and *Trachurus* spp. in the local food web. To better depict their role, stomach content and stable isotope analyses were performed on specimens caught during routine acoustic surveys carried out along the western Adriatic coast. The results show that the two *Trachurus* species (*T. trachurus* and *T. mediterraneus*) share a similar diet but present spatial segregation along a latitudinal (i.e., thermal) gradient, while the two *Scomber* species (*S. scombrus* and *S. colias*) differ for prey preferences and present spatial segregation too, along a bathymetric gradient. The positions (trophic niches) of these species in the food web only partially overlap. This fact allows good resource partitioning and the coexistence of these species in the Adriatic Sea, limiting the risk of possible future collapses of some of these fish populations, with inevitable cascade effects on the entire marine food web.

**Abstract:**

The Mediterranean is one of the most overfished seas of the world where mesopredators are severely threatened. The trophic strategies of four pelagic species that inhabit the Adriatic Sea (*Scomber* spp. and *Trachurus* spp.) were investigated through an integrated approach of stomach contents and stable isotopes analyses. Our study demonstrated that *Scomber colias* feeds mainly on strictly pelagic prey, with fish larvae as a secondary prey in the Southern Adriatic Sea, while *S. scombrus* feeds on prey belonging to higher trophic levels. Smaller specimens of *Trachurus mediterraneus* have a diet mainly based on pelagic prey, while larger fishes rely on prey such as benthic decapods, showing an ontogenetic shift in the diet of the species. *Trachurus trachurus* shows a preference for offshore and deeper areas and a diet such as that of its congeneric, but no clear ontogenetic shift was observed. This spatial segregation allows the co-existence of these two species of *Trachurus*. *Scomber colias* mainly inhabits southern areas and *S. scombrus* shows a preference for the northern sectors. This latitudinal gradient avoids the overlap of their trophic niches. Bayesian mixing models confirmed that the trophic niches of these species only partially overlap in the middle of the trophic web.

## 1. Introduction

Pelagic fishes are usually associated with the upper section of the water column and with open ocean environments [1]. Pelagic fishes vary in form and function, ranging from small forage fishes, such as anchovies and mackerels, to large predator piscivorous fishes, such as tuna and sharks [1]. From an ecological point of view, small/middle-sized pelagic fishes such as *Trachurus* spp. and *Scomber* spp. occupy a particular trophic level between the phyto-zooplanktivorous pelagic fishes (e.g., sardines and anchovies) [2] and the large piscivorous species (e.g., tuna and related species) [3]. They play an important trophic role as mesopredators. Consequently, their global biomass is typically smaller than basal small pelagics (sardines and anchovies) and larger than the top pelagic predators [4]. Small pelagic fishes share some common characteristics: several length classes and cohorts inside a population, an elevated trophic plasticity, high gregariousness, swimming capability and resistance [5].

Pelagic fishes belonging to the genera of *Scomber* and *Trachurus* are not a target for most of the Mediterranean and Black Sea fisheries due to their lower commercial value compared to other pelagic species, such as herrings, anchovies, and sardines, that together represent more than 50% of the total amount of landings [6,7,8] and are, thus, referred to as ‘ancillary’. In the period from 2016 to 2018, *Scomber* spp. and *Trachurus* spp. represented ca. 4% of the landings [8]. Despite few data being available for these species, they can certainly represent a crucial economical resource for small-scale and local fisheries.

Our study focused on four species found in the Adriatic Sea: the Atlantic chub mackerel (*Scomber colias*), the Atlantic mackerel (*S. scombrus*), the Mediterranean horse mackerel (*Trachurus mediterraneus*) and the Atlantic horse mackerel (*T. trachurus*).

The Adriatic Sea faces high fishing pressure and both chub and horse mackerels are targets of multiple fisheries here [9]. Chub mackerel is traditionally captured by purse seiners [10]. The population of *S. scombrus* from the north and central Adriatic Sea showed a marked decline from the 1990s to 2010 of maximum age and total length that could be related to overexploitation [11]. Moreover, a clear decline in catches in the last two decades has been highlighted [12,13]. Since *S. scombrus* represents 89.2% of the total scombrid landings and considering that *S. colias* is often sold as *S. scombrus*, it is difficult to obtain reliable landings data on these two species, separately [14]. Mackerels are mainly harvested with purse-seine, pelagic and mid-water trawls, and poorly with gill and trammel nets. Landings data are also scarce for horse mackerels, even if they are the object of pelagic (purse-seines and mid-water trawls) and demersal fishing in the Adriatic Sea [15].

*Scomber scombrus* is a boreal fish that inhabits the Atlantic Ocean at temperate and boreal latitudes. Although less abundant, the species is also present in the Mediterranean and Black Sea, more commonly in their coldest sectors. *Scomber colias* is a widespread sub-tropical species that is very common in the warmer areas of the Atlantic Ocean and in the warmest sectors of the Mediterranean and Black Seas [16].

*Trachurus mediterraneus* can be frequently found along the eastern coast of the Atlantic Ocean only in the northern hemisphere; it is widespread in all the Mediterranean basin, very common in the Adriatic basin and less common in the Black Sea. *Trachurus trachurus* is distributed all along the eastern coast of the Atlantic Ocean from Norway and Iceland to the Cape Verde Islands, and in the Mediterranean and Black Seas [17].

Resource partitioning, trophic relationships, and prey–predator relationships in marine environments are increasingly studied through an integrated approach using the more recent stable isotopes analysis (SIA) and the traditional stomach contents analysis (SCA) [18]. For better knowledge of marine trophic webs’ structures, the most used isotopes are nitrogen *δ*^15^N (^15^N/^14^N) and carbon *δ*^13^C (^13^C/^12^C) [19,20,21].

Stable nitrogen isotope value (*δ*^15^N) is a proxy of the trophic level of a species, so it can be used to identify the position of a species within a trophic food web [19,20]. The stable carbon isotope value (*δ*^13^C) is more helpful to determine the origin of food sources for an organism [19,22] and to discriminate between a benthic or a pelagic origin of food, or continental vs. marine inputs [23]. The difference in *δ*^15^N and *δ*^13^C between consumers′ tissues and their diet is termed Trophic Enrichment Factor (TEF), and it corresponds to values between 2.5 and 5‰ for *δ*^15^N and <1‰ for *δ*^13^C [19,20,24]. For this reason, nitrogen is a better proxy of the trophic position of a species than carbon.

A stomach contents analysis reflects the food ingested in a unique point or a few points in space and in a restricted time, due to the fast turnover times of gut contents. Therefore, it provides only a snapshot of the species′ dietary habits, showing their last food intakes. Moreover, some specimens can present an empty stomach. This is why the SCA may offer a snapshot of the diet of an individual in a precise time and space, while the SIA provides time-integrated information, especially if it is run on low turn-over rate tissues, such as muscles [20,21,25,26]. With this integrated dual approach, mechanisms of resource partitioning, prey–predator relationships and trophic dynamics of energy flows inside food webs can be better understood or even totally overturned. At the Mediterranean level, there are gaps in the knowledge on feeding habits and resource partitioning among middle-sized pelagic fishes, such as mackerels and horse mackerels.

The main goal of this study is to increase our knowledge on the trophic ecology of these four co-existing “ancillary” species, considering possible ontogenetic shifts, analyzing mechanisms of resource partitioning and diet’s environmental drivers. Consequently, we will better highlight their role in the pelagic food web as mesopredators and propose new hypotheses on their importance in the Adriatic Sea food web.

## 2. Materials and Methods

### 2.1. Study Area and Sampling

The Adriatic Sea is conventionally divided into three sub-basins: the North Adriatic, Central Adriatic and South Adriatic [27].

Samples were collected between 1 June and 15 July 2019, during the GSA 17 and GSA 18 acoustic surveys targeting small pelagic fish, conducted on board R/V G. Dallaporta [28] in the framework of the MEDiterranean International Acoustic Survey (MEDIAS) project (http://www.medias-project.eu/medias/website/, accessed on 6 October 2022) [29], within the EU Fisheries Data Collection Framework (DCF). During this scientific survey, an acoustic sampling was performed according to standardized methodology, using a SIMRAD EK80 split-beam multi-frequency echosounder (at 38, 70, 120 and 200 kHz frequencies, KONGSBERG SIMRAD, Kongsberg, Norway) installed on board the research vessel. The covered parallel transected perpendicular to the coastline [30], started from a 10 m depth when possible, and reached a 200 m depth or the Adriatic Midline in shallower regions [31]. The maximum depth was set at 200 m, i.e., about the depth of the edge of the continental shelf, where the abundance of most small pelagic fish decreases [28]. Simultaneously, pelagic fishes were collected through a pelagic trawl net (10 m vertical opening and 12 m horizontal opening, with 18 mm mesh size) equipped with a wireless SIMRAD “trawl eye” system (KONGSBERG SIMRAD, Kongsberg, Norway) that allowed information to be gathered on the correct opening of the net and on entering fishes during trawling. The hauls′ standard duration was around 30 min. Sardine, anchovy and sprat were the main targets, but some hauls were characterized by the presence of other pelagic fish species that were identified, collected and grouped for each haul under the name of OPS (Other Pelagic Species), such as mackerels (*S. colias* and *S. scombrus*) and horse mackerels (*T. mediterraneus* and *T. trachurus*) [29]. All collected OPS samples were immediately frozen on board at −20 °C.

**Figure 1:**
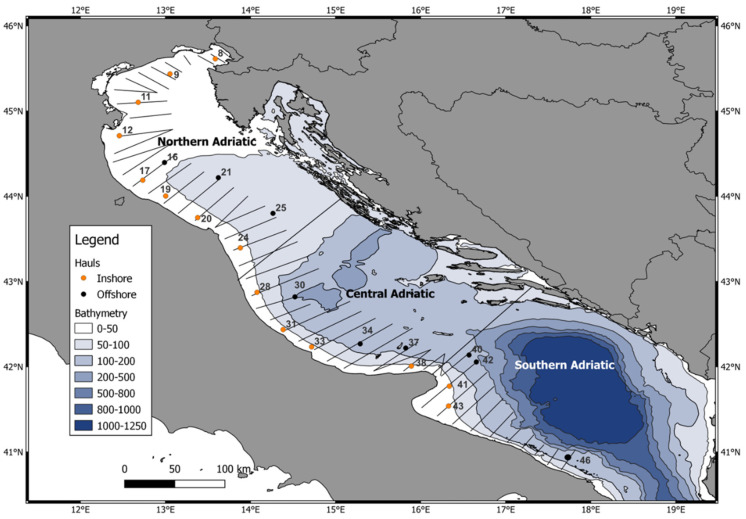
Surveyed subareas and the selected hauls (in orange the inshore hauls and in black the offshore hauls).

A sub-set of hauls was selected to cover the whole studied area; this was divided into three different geographic sectors or subareas considering bathymetric and river runoff characteristics (Figure 1; Supplementary Table S1) In this study, according to sub-basin features, we divided the Adriatic Sea into 3 areas: North (hauls 8–24, N), Central (hauls 28–38, C) and South Adriatic (hauls 40–46, S). Furthermore, we also divided the hauls into inshore (<40 m depth, hauls carried out during daytime) and offshore hauls (≥40 m depth, hauls carried out during the night).

Once the samples were at the laboratory, each specimen was labelled with an identification code and weighed (wet weight, WW, in g). The total length, TL, in cm, was measured with an icthyometer (±0.1 cm). In adult individuals, the gonads were observed to determine sex and maturity stage according to the maturity scale of [32], which is commonly used for mackerels and recommended for horse mackerels [33]. Otherwise, individuals were classified as undetermined (ND). Carefully extracted stomachs were preserved at 4 °C with 70% ethanol. Then, a portion of muscle close to the dorsal fin was dissected [34,35] and stored at −20 °C in sterile test tubes for SIA.

### 2.2. Stomach Content Analysis

Each stomach was extracted and preserved with 70% ethanol. For each stomach, fullness was calculated as a proxy of feeding intensity:

Stomach fullness (%) = stomach content weight/body weight × 100

Using a stereomicroscope (Zeiss STEMI 2000), stomach contents were sorted by high taxonomic level, stored in 70% ethanol, and then identified to the lowest taxonomic level possible [25]. To give an idea of the level of digestion of the contents, a value from 0 (undigested) to 3 (highly digested) was assigned to each content [36]. Each stomach content was then weighted using an analytical balance. Moreover, the traditional trophic indices were estimated as:

(i) Percentage of frequency of occurrence (%*F*): $\%F=n\times 100N^{-1}$ where n is the number of guts containing a certain prey and *N* is the total number of guts examined.
(ii) Percentage of gravimetric composition (%*W*): $\%W=w^{i}\times 100W_{p}{}^{-1}$ [37], where *w^i^* is the total weight of individuals of a certain prey *i* and *W_p_* is the total weight of prey items.
(iii) The index of relative importance (%*IRI*): %*IRI* = *IRI*/*∑IRI* × 100, where *IRI* = (%*N +* %*W*) × %*F*, as described in [38].
(iv) Diet diversity within the year was calculated for each sex based on the Shannon–Wiener H′ index, calculated as H’ = −∑*p_i_* × ln*p_i_*, where *p_i_* is the ratio between the number of individuals (e.g., density) of each prey items (the *i*th species) and *N* is the total number of individuals of all prey species found in the stomach contents (*p_i_ = n_i_*/*N*).

### 2.3. Stable Isotope Analysis

For each area and for each of the four species collected within that area, at least 5 specimens (when available) for each of three length categories established based on frequency–length distribution (small, medium and large—see Results section) were selected. The samples were oven-dried for 24 h at 60 °C [25], then converted to a fine powder with a mortar and pestle; ca. 0.8–1.3 mg was weighed with an analytical balance and placed into tin capsules for subsequent analyses (Elemental Microanalysis Tin Capsules Pressed, Standard Weight 5 × 3.5 mm) [25].

Carbon and nitrogen contents were determined through an elemental analyzer (Thermo Flash EA 1112, Thermo Fisher Scientific Inc., Waltham, MA, USA) for the determination of total carbon and nitrogen, and then analyzed for *δ*^13^C and *δ*^15^N in a continuous-flow isotope-ratio mass spectrometer (Thermo Delta Plus XP, Thermo Fisher Scientific Inc., Waltham, MA, USA) at the Laboratory of Stable Isotopes Ecology of the University of Palermo (Italy). The stable isotope ratio was expressed in relation to international standards (atmospheric N_2_ and PeeDee Belemnite for *δ*^15^N and *δ*^13^C, respectively), as:

*δ*^13^C or *δ*^15^N = [(R_sample_/R_standard_) − 1)] × 10^3^

where R = ^13^C/^12^C or ^15^N/^14^N. Analytical precision based on standard deviations of internal standards (International Atomic Energy Agency IAEA-CH-6; IAEA-NO-3; IAEA-N-2) ranged from 0.10 to 0.19‰ for *δ*^13^C and 0.02 to 0.08‰ for *δ*^15^N.

Since the presence of lipids can alter the values of *δ*^13^C [39,40], samples with high lipid concentration can be defatted to avoid ^13^C depletion. However, lipid extraction can alter *δ*^15^N values, complicate sample preparation and reduce samples availability, which is a crucial point when analyzing small animals. The C/N ratio was used as a proxy of lipid content, because their values are strongly related in animals [39]. In particular, the normalization was applied to samples with a C/N ratio > 3, according to [39]: *δ*^13^C_corrected_ = *δ*^13^C_untreated_ − 3.32 + 0.99 × C/N_bulk_, and the *δ*^13^C_corrected_ value was used for consecutive analyses.

Correlation among TL and, separately, variations in values of *δ*^13^C and *δ*^15^N was tested using R [41] with the function “cor.test”.

### 2.4. Data Treatment

Permutational Multivariate Analysis of Variance (PERMANOVA) main tests were carried out considering two statistical designs:

(1) A two-factors nested design for determining interspecific differences:

- Species as a fixed factor with four levels (*S. colias*, *S. scombrus*, *T. mediterraneus* and *T. trachurus*).

- Area as a random factor (nested in species, due to the lack of some area levels) with three levels (North, Central and South Adriatic).

(2) A one-factor design with area as a fixed factor with three levels for determining intraspecific differences.

We did not consider the inshore or offshore position as a factor in our statistical analyses, since we did not have enough specimens for each species from each area that allowed us to construct a symmetrical design with fixed factors, and to draw conclusions on the depth and diel variation of the trophic behavior of the species. The permutation method was “permutation of residuals under a reduced model”, with 9999 permutations in the first case, while we used an “unrestricted permutation of raw data” method in the second case. Differences were considered significant when *p*-values resulted <0.05, both for univariate and multivariate analyses.

Differences among the values of stomach fullness were tested on a Euclidean distance resemblance matrix of untransformed data.

Investigations on the diets were conducted considering prey biomasses as variables. We did not consider the “other” items to carry out these analyses. Multivariate analyses were conducted on a Bray–Curtis distance resemblance matrix of log(x + 1)-transformed biomass data) [42]. Factors found to be significant in the investigation of the diets underwent CAP (Canonical Analysis of Principal Coordinates [43]) analyses, to visualize samples separation on the basis of putative factors. The PERMDISP multivariate dispersion test was conducted on stomach contents biomasses to evaluate the homogeneity of multivariate dispersions between species based on resemblance measures. PERMDISP mean values can be considered as indicators of diet generalism vs. specialism. A SIMPER (Similarity Percentage) analysis was performed to obtain the percentage contribution of different prey to the average similarity of diet among samples in each area. The analysis was conducted using the Bray–Curtis similarity matrix with a cut-off of percentage contribution at 60%.

Furthermore, to assess the trophic diversity of each species in each area, the Shannon–Wiener index (H’) was calculated for each sample and univariate PERMANOVA analyses were performed with the same design and method used for stomach fullness.

Univariate and multivariate analyses were conducted on the SIA results obtained for the four species. PERMANOVA main tests were carried out on Euclidean distance resemblance matrices of untransformed *δ*^13^C and *δ*^15^N values separately, and of these two variables selected together to detect significant differences among the levels of the fixed factor “Species” and of the random factor “Area” nested in “Species”. In this case, to detect differences among the four taxa, we conducted pairwise comparisons if *p* < 0.05 for the factor species.

The SIBER package (Stable Isotope Bayesian Ellipses in R) [44] was used to determine the isotopic niche width of each species and their trophic preferences. We carried out analyses with SIBER two times (Table 1). In the first case, we considered 4 ‘communities’, each corresponding to one species. In the second case, we considered 4 communities, each corresponding to one species, and we included three groups in each community, each corresponding to the sub-area of collection.

SIBER was used to calculate the total area of the convex hull (TA), the corrected Standard Ellipse Areas (SEA_C_), and the mean distance to centroid (CD), *δ*^15^N range (NR), *δ*^13^C range (CR), mean nearest neighbor distance (MNND) and standard deviation of the nearest neighbor distance (SDNND) [45]. The TA gives an indication of the variety of food sources on which the species can feed, while SEA_C_ (which contains approximately 40% of the data within a set of bivariate data) represents the core area for a population or community [44,45]. CD estimates trophic diversity within a food web and is a function of the degree of group spacing. NR gives information on the trophic length of the community; CR provides an estimate of the diversity of basal resources; MNND provides a measure of density and clustering of species within the community; and SDNND gives a measure of the evenness of spatial density and packing [44].

## 3. Results

### 3.1. Sampling Data

A total of 62 individuals of *S. colias* were sampled (Table 2); most were juveniles. Sixteen individuals of *S. scombrus* were captured only at inshore positions. Most of the 93 specimens of *T. mediterraneus* were adults captured mainly in the North and Central Adriatic Sea. Forty-two individuals of *T. trachurus* were sampled, mainly juveniles caught in the Central Adriatic.

### 3.2. Condition Indexes and Fullness of “Ancillary” Pelagic Fishes

**Figure 2:**
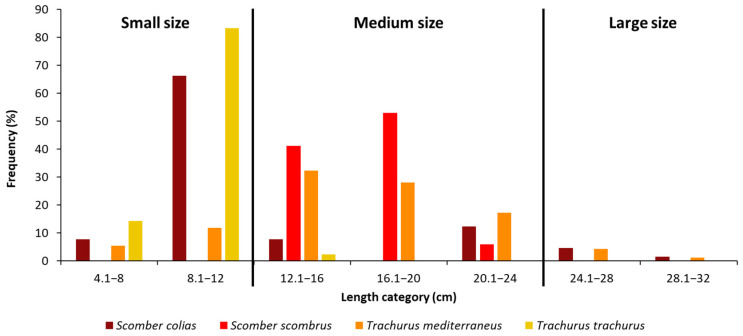
Length frequency distributions of n = 62 individuals of *Scomber colias*, n = 16 individuals of *Scomber scombrus*; n = 93 individuals of *Trachurus mediterraneus* and n = 42 individuals of *Trachurus trachurus* captured during summer in the Adriatic Sea. Specimens were divided into three size classes according to their length: small (<12 cm TL), medium (from 12.1 to 24 cm TL) and large (≥24 cm TL) size.

In general, the TL values ranged between 4.1 and 30.8 cm. To facilitate our analyses, we set 3 size-class categories: small, medium and large size (Figure 2). More than half of the captured specimens of *S. colias* had a TL between 8.1 and 12 cm. Most of the specimens of *S. scombrus* belong to the medium class size, with a TL between 12.1 and 20 cm (93% of the total). The mainly represented length classes for *T. mediterraneus* were the two between 12.1 and 20 cm (Figure 2). Eighty percent of the specimens of *T. trachurus* had a length of 8.1–12 cm.

**Figure 3:**
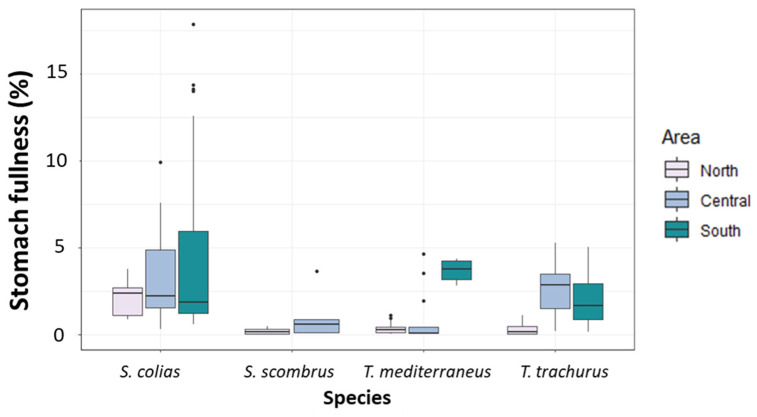
Boxplot of stomach fullness (%) measured in the specimens of the four “ancillary” species captured in the North, Central and South Adriatic Sea.

The PERMANOVA main test revealed no significant differences among the values of stomach fullness measured in specimens of *S. colias* (Figure 3 and Supplementary Tables S2 and S3). For *S. scombrus*, no significant differences were detected among the two sub-areas of collection (Figure 3 and Supplementary Tables S2 and S3). Among the specimens of *T. mediterraneus*, the highest mean value of stomach fullness was found in the South Adriatic (Figure 3 and Supplementary Table S2), and differences among areas were significant (*p* < 0.001, Supplementary Table S3). Among *T. trachurus* specimens, the highest mean value of stomach fullness was found in the Central Adriatic (Figure 3 and Supplementary Table S3). Values of stomach fullness in this last species were significantly different among the different areas (*p* < 0.05, Supplementary Table S3).

### 3.3. Diets of “Ancillary” Pelagic Fishes

#### 3.3.1. *Scomber colias*

**Figure 4:**
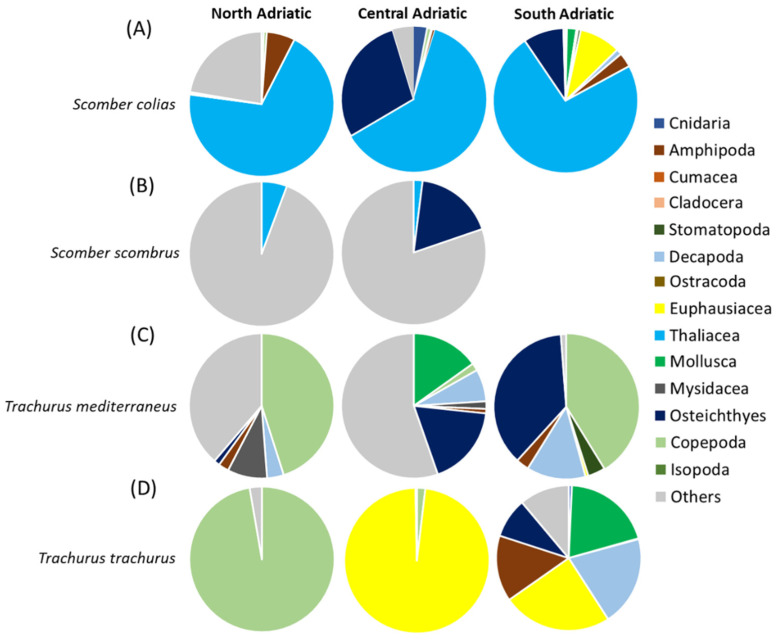
Composition expressed as %IRI of the diets of (**A**) *Scomber colias*, (**B**) *Scomber scombrus*, (**C**) *Trachurus mediterraneus* and (**D**) *Trachurus trachurus* in the North, Central and South Adriatic Sea. The category of “other material” includes undigested material, fish scales and parasites.

A total of 39 taxa were identified (listed in Supplementary Table S4). In terms of %IRI (Figure 4A), the main prey was represented by thaliaceans, particularly Salpidae, in all the areas. Several Osteichthyes were also found, especially in the Central and Southern Adriatic Sea, with *Engraulis encrasicolus* larvae as the main prey. Crustaceans were mainly represented by several species of amphipods in all areas, and euphausiids, the latter only in the South Adriatic. Prey of secondary importance were sepiolid cephalopods, Calycophorae hydrozoans and Decapoda larvae. The “Others” prey category, which was of noticeable importance in the North Adriatic, was represented by scales and Digenea Trematoda parasites (Figure 4A and Supplementary Table S4). The PERMANOVA main test carried out on the diet composition (%W) of *S. colias* showed significant differences for the factor of area (*p* < 0.05, Supplementary Table S5). The SIMPER results showed an average dissimilarity of 92% between the diet of *S. colias* in the North and Central Adriatic and of 87% between the diets in the Central and South Adriatic (Supplementary Table S6).

Values of H’ differed significantly among the areas (*p* < 0.01, Supplementary Table S7), with the highest value in the offshore South Adriatic.

#### 3.3.2. *Scomber scombrus*

Eight taxa were identified (listed in Supplementary Table S8). In terms of %IRI (Figure 4B), the most representative contents category was “Others” in both areas, represented mostly by scales and Digenea Trematoda parasites. Parasites were more present in the northern subarea, while scales were more abundant in the southern one. Clupeiformes and fish skeletons were also important prey items, but only in the Central Adriatic. The main prey for both areas was thaliaceans such as *Pyrosoma* sp. in the North and Salpidae specimens in the Central Adriatic. The PERMANOVA main test carried out on the diet composition (%W) of *S. scombrus* did not show significant differences for the factor of area (Supplementary Table S5). The SIMPER analysis on diet composition showed an average dissimilarity of 100% between the diet of *S. scombrus* in North and Central Adriatic (Supplementary Table S9).

Values of H’ did not show significant differences (Supplementary Table S7).

#### 3.3.3. *Trachurus mediterraneus*

In the 77 full stomachs analyzed, a total of 44 taxa were identified (listed in Supplementary Table S10). In terms of %IRI (Figure 4C), the most representative prey were pelagic copepods (mainly *Acartia* and *Euchaeta* spp.) in the North and in the South Adriatic. Many scales were found in stomach content in the North and Central Adriatic. Several *E. encrasicolus* larvae were also found, mainly in the Central Adriatic. Amphipods were quite representative, with some benthic (Gammaridea) and pelagic (Hyperiidea) taxa. Benthic decapods such as *Alpheus glaber* and *Philocheras bispinosus* were also present, as well as mysids such as *Siriella* sp. (above all in the South Adriatic). Prey of secondary importance were small bivalves, gastropods, stomatopods and Foraminifera species. The PERMANOVA main test carried out on the diet composition (%W) of *T. mediterraneus* did not show significant differences (Supplementary Table S5). The SIMPER results showed an average dissimilarity of 98% between the diet of *T. mediterraneus* in the North and Central Adriatic and of 94% between the diets in the Central and South Adriatic (Supplementary Table S11).

Values of H’ did not show significant differences among the levels of the factor of area (Supplementary Table S7).

#### 3.3.4. *Trachurus trachurus*

Out of 42 analyzed stomachs, 34 were full. Twenty-two taxa were identified (see Supplementary Table S12). In terms of %IRI (Figure 3D), in the North Adriatic, the most representative prey were copepods (mostly *Acartia* sp.), whereas in the Central Adriatic, the euphausiid *Nyctiphanes couchii* was the most important prey. In the South Adriatic, crustaceans such as the copepods *Euchaeta* sp., *Calanus* spp. and hyperiids were also abundant. Osteichthyes and Mollusca (the species *Creseis acicula*) were prey of secondary importance, occurring only in the South Adriatic. The PERMANOVA main test carried out on the diet composition (%W) of *T. trachurus* showed significant differences among the levels of the factor “Area” (*p* < 0.01; Supplementary Table S5). The SIMPER results showed an average dissimilarity of 100% between the diet of *T. trachurus* in the North and Central Adriatic and of 91% between the diets in the Central and South Adriatic (Supplementary Table S13).

Values of H’ did not showed significant differences among the levels of the factor “Area” (*p* < 0.001, Supplementary Table S7).

### 3.4. Overlap of the Diet of “Ancillary” Pelagic Fishes

Values of stomach fullness and diet diversity did not differ for the factor “Species” but were significantly different when considering the factor “Area” nested in the factor “Species” (*p* < 0.001, Supplementary Tables S3 and S7). The PERMANOVA main test showed that the composition in terms of %W of the diets and the diet diversity (H’) of these species were significantly different for the factor “Area” nested in the factor “Species” (*p* < 0.001, Supplementary Tables S5 and S7).

SIMPER showed that the stomach contents of the two species of the genus *Scomber* had an average dissimilarity of 98% (mainly justified by the contributions of Salpidae, fish skeletons and larvae of *Engraulis encrasicolus*), while those of the two *Trachurus* species had an average dissimilarity of 99% (mainly justified by the contributions of *N. couchii*, fish skeletons and *Siriella* sp.) (Supplementary Table S14).

The PERMDISP pair-wise test (Supplementary Table S15) showed that dispersions were significant among *Trachurus* species and among *Trachurus mediterraneus* and *Scomber* species. Considering “means and standard errors”, a more generalized diet was noticeable for *Trachurus mediterraneus*. On the contrary, *Scomber scombrus* resulted in the species with the most specialized diet.

### 3.5. Stable Isotope Composition of “Ancillary” Pelagic Fishes

**Figure 5:**
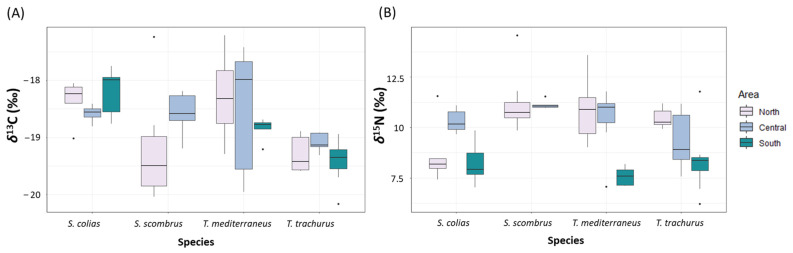
Boxplot showing the values of (**A**) *δ*^13^C (‰) and (**B**) *δ*^15^N (‰) measured in the four “ancillary” species captured in the North, Central and South Adriatic Sea.

The mean values of *δ*^13^C ranged between −19.4 ± 0.4‰ and −18.2 ± 0.4‰. The mean values of *δ*^15^N ranged between 8.2 ± 0.9‰ and 10.9 ± 1.3‰ (Figure 5 and Supplementary Table S16). The values of *δ*^13^C significantly differed for the factor of species (*p* < 0.05, Supplementary Table S17). The values of *δ*^15^N differed significantly for the factor of area nested in the factor of species (*p* < 0.001, Supplementary Table S17). Multivariate analyses on the values of both *δ*^13^C and *δ*^15^N showed significant differences for the factor of area nested in the factor of species (*p* < 0.001, Supplementary Table S17). The pair-wise tests showed significant differences between the values of *δ*^13^C of *T. trachurus* and *T. mediterraneus* (*p* < 0.05), and between the values of *δ*^13^C of *S. colias* and *T. mediterraneus* (*p* < 0.01, Supplementary Table S17).

Correlation between the values of *δ*^13^C and TL resulted to be significant for *S. colias* and for *T. mediterraneus*, with higher values of *δ*^13^C with increasing TL (*p* < 0.01 and R^2^ = 0.39, *p* < 0.001 and R^2^ = 0.4, respectively) (Supplementary Figure S1).

Correlation between the values of *δ*^15^N and TL resulted to be significant for *S. scombrus* and for *T. mediterraneus*, with higher values of *δ*^15^N with increasing TL (*p* < 0.01 and R^2^ = 0.45, *p* < 0.001 and R^2^ = 0.56, respectively).

### 3.6. Tropic Niches of “Ancillary” Pelagic Fishes

**Figure 6:**
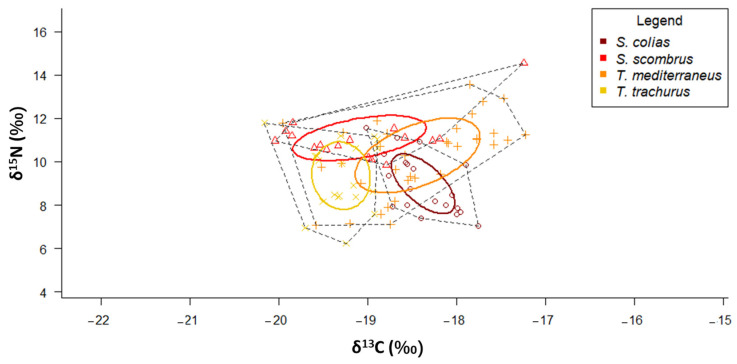
*δ*^13^C-*δ*^15^N scatterplot with standard ellipses corrected for small sample size population (SEA_C,_ ‰^2^) overlaid for the specimens of the four “ancillary” species collected in the Adriatic Sea.

According to the SIBER results, *T. mediterraneus* showed the widest SEA_C_, while *S. colias* the narrowest one (Figure 6 and Table 2). The SEA_C_ of *S. scombrus* and *T. mediterraneus* are stretched along the x-axis, while those of *S. colias* and *T. trachurus* are stretched along the y-axis. *S. scombrus* shows the lowest values of both MNND and SDNND (Table 3), while *T. mediterraneus* shows the highest ones.

According to the SIBER results, *T. mediterraneus* caught in the Central Adriatic shows the widest SEA_C_, while *S. colias* caught in the Central Adriatic shows the narrowest one (Table 3 and Supplementary Figure S2).

## 4. Discussion

### 4.1. Overall Feeding Ecology of the Pelagic “Ancillary” Species

For the first time, this study provides data on the resource partitioning of four small “ancillary” pelagic species in the Adriatic Sea by using an integrated approach of stomach contents and stable isotopes analyses.

#### 4.1.1. *Scomber colias*

The SCA allowed us to confirm that *Scomber colias* mainly feeds on prey captured within the water column, such as gelatinous plankton (thaliaceans), fish larvae, euphausiids and amphipods, as demonstrated by other studies conducted in the Aegean Sea [46] and in the Eastern Atlantic Sea [47,48]. Although other studies found mysids to be an abundant item in the Canary Islands [47] and in the Eastern Adriatic [10], they were absent in *S. colias*’ stomachs from our sampling area. Since the specimens were captured at the beginning of summer, our results could be inferred by the fact that, in this period, thaliaceans and hydrozoans blooms are frequent [49].

The diet diversity of this species was the highest in the South Adriatic. Considering that higher depths found in this sector of the basin guarantee access to a greater number of food items, a larger trophic diversity could be correlated to a wider distribution of *S. colias* specimens throughout the water column. In this sector, *Engraulis encrasicolus* larvae were the secondary prey for chub mackerels. Moreover, euphausiids that migrate to the surface during diel vertical migrations [50] are important prey in the South Adriatic for *S. colias*. The SIA showed a slight decrease in *δ*^15^N values with size. This could be related to the higher consumption on euphausiids that are mostly represented as primary consumers and omnivores of low trophic levels [51,52]. Accordingly, the increase in *δ*^13^C values with size indicated a displacement of large specimens in deeper areas (offshore or in the southern sector of the Adriatic basin).

Overall, *S. colias* is confirmed to feed almost in the pelagic compartment and on low trophic level organisms, with a good intraspecific resource partitioning driven by a physical separation of specimens according to their size, and with juveniles mostly inhabiting coastal waters and adults living in offshore ones.

#### 4.1.2. *Scomber scombrus*

A few individuals of *S. scombrus* were captured in inshore areas and only in the North and Central Adriatic. In the South Adriatic, trophic diversity was slightly higher, mainly due to the presence of bony fishes and thaliaceans, which are also reported to concur with *S. scombrus*’ diet in the Bay of Biscay [53,54], in the Mediterranean Sea [55], and in the northeast Atlantic [56]. Both isotopic signatures increased with increasing size, with *δ*^15^N that showed a higher mean value in the Central Adriatic, probably due to both a diet shift with increasing size and the preference of larger specimens for deeper areas.

These results also support that *S. scombrus* feeds almost exclusively on pelagic prey and most likely on higher trophic levels with increasing sizes. Notwithstanding this evidence, further studies are needed to confirm or refute an intraspecific resource partitioning based on differential spatial distribution according to size.

#### 4.1.3. *Trachurus mediterraneus*

*Trachurus mediterraneus* mainly ingested copepods, mysids, amphipods and decapods. These results are like those obtained for specimens collected in the eastern Adriatic Sea [15,57]. Contrarily to the same results reported in the literature, euphausiids and polychaetes did not contribute to the Mediterranean horse mackerel’s diet in the western Adriatic Sea. Conversely, we frequently found several benthic prey, such as gammarid amphipods, benthic decapods, stomatopods, bivalves and gastropods, in stomach contents, according to findings from the Black Sea [58]. Interestingly, in the South Adriatic, most of the contributions to the diet were made by pelagic prey, probably because, due to the high depth of this sub-area, the search for food is limited in the upper part of the water columns. In the North and South Adriatic, the most important prey was Copepoda, but their species composition was totally different, according to mesoscale variations in zooplanktonic communities [59]. The presence of parasites characterized the stomachs of specimens collected in the North Adriatic, as was previously shown in the literature [60]. The isotopic signatures of ***δ***^13^C and ***δ***^15^N significantly increased with size, showing an ontogenetic shift in the diet, with larger animals preferring the pelagic prey of higher trophic levels. Since carbon enrichment in fishes tends to be associated with variations in the baseline of the trophic chain [22], these findings confirm that in *T. mediterraneus*, the intraspecific partitioning of trophic resources is granted by an ontogenetic diet shift.

#### 4.1.4. *Trachurus trachurus*

The results of the SCA showed that the diet of *T. trachurus* deeply varied among the three areas, with copepods mostly contributing to its diet in the North Adriatic and euphausiids being dominant in the Central Adriatic. The Central Adriatic showed the highest stomach fullness value, with the euphausiid *Nyctiphanes couchii* being the most frequent prey, as also observed in the eastern Central Adriatic Sea [61]. Higher depths in this basin favor the diel vertical migrations of this species. In the South Adriatic, euphausiids, hyperiids, molluscs (the pelagic snail *Creseis acicula*), fish larvae and decapods contributed together to the diet of this species, according to other studies conducted in the Adriatic [62] and in the Strait of Sicily [35]. In those studies, many benthic or suprabenthic prey were found, but these items were absent in the stomachs we analyzed. The highest trophic diversity was found in the South Adriatic, probably because, as for *S. colias*, higher depths enhance the probability of catching a greater variability of prey in the water column.

Mainly juvenile specimens of the Atlantic horse mackerel were collected, but *δ*^15^N values slightly increased with size, pointing out to a possible shift in the diet with increasing size. Otherwise, *δ*^13^C values showed an almost constant trend, suggesting that at least specimens of the size range explored in this study mostly remain in the same foraging area without great inshore–offshore displacement and/or change in food source (pelagic vs. benthic).

The specimens of *T. trachurus* we collected fed almost exclusively on pelagic items but, conversely to *T. mediterraneus*, an intraspecific resource partitioning cannot be confirmed by the results of this study.

A general comment could be made on the relevant presence of fish scales in the stomach contents of specimens collected in the Central Adriatic, except for *S. colias*. This could be related to fisheries discards, the amount of which is high in this area of the Adriatic Sea [6] and could represent an easily accessible food. Since scales were ingested by most of the species we investigated in this study and since the Northern-Central Adriatic Sea is one of the most intensively fished areas of Europe—a kind of trawling hot spot [63]—it is probable that scales are not the remains of the digestion of actively captured fish but are instead directly ingested by chub and horse mackerels while scavenging on this huge amount of dead fishes.

### 4.2. Resource Partitioning of the Pelagic “Ancillary” Species

The results obtained by the SCA and SIA highlighted resource partitioning among the four pelagic “ancillary” species.

Focusing on the two *Scomber* species, clear trophic niche segregation is achieved by the fact that, although the two species feed mostly on pelagic prey, *S. colias* specimens captured for this study (predominantly juveniles) prefer low trophic level organisms while *S. scombrus* feeds on higher trophic level prey. Moreover, these two species face spatial segregation, with *S. colias* inhabiting mainly southern areas and *S. scombrus* showing a preference for the northern sectors, as it happens along the Portuguese coasts following a decreasing gradient of water temperatures [64]. According to the SIBER results, the food web length of *S. colias* is greater than that of *S. scombrus*, pointing to multiple basal carbon sources with larger animals that feed on organisms of higher trophic levels. Low values of MNND and SNND help to infer a relative intraspecific trophic redundancy that was higher in *S. scombrus*. These results seem to be in contrast with those of SCA, but since we did not consider the size as a factor for running SIBER, it is likely that the intraspecific resource partitioning is driven by a differential spatial distribution according to the size. Further studies that allow the collection of a higher number of specimens of Atlantic mackerels will allow size to be considered as a factor for discussing the SIA results.

Juveniles of *Trachurus* mainly base their diet on pelagic prey, in agreement with what was previously described in [57] for the two species in the Adriatic Sea. Interspecific overlap is avoided by spatial segregation of the two species, since *T. mediterraneus* is located preferentially in the North and Central Adriatic, while *T. trachurus* specimens are relegated to the southern and deeper part of the Adriatic. This bathymetric segregation was already observed for the two species in the southern Sicilian waters, with Mediterranean horse mackerels occupying more shallow habitats than Atlantic horse mackerels [65]. Among the two species, only *T. mediterraneus* showed an ontogenetic shift in its diet, with larger specimens captured inshore (and during daytime) that rely more on benthic decapods. Concerning the SIBER results, *T. mediterraneus* had the longest food web length among all the four investigated species. There was a low trophic redundancy for both species, as confirmed by MNND values. An even distribution of trophic niches was only displayed by *T. trachurus*, probably because this species displays an intraspecific resource partitioning driven by factors not considered in this study.

Considering all the four species together, *T. mediterraneus* displayed the widest SEA_C_, while *S. colias* displayed the narrowest one. The latter species also displayed the lowest mean value of trophic diversity, but these results should be carefully considered, since few specimens of Atlantic chub mackerel and predominantly juvenile were taken for this study.

Diet generalism vs. specialization can be interpreted as a resource partitioning indicator [25]. *Trachurus mediterraneus* and *S. colias*, respectively, showed a more generalized diet when compared to their congeneric species, and this was supported by the results of both the SCA and SIA. PERMDISP pairwise tests and CD values showed that *T. mediterraneus* has the most diversified diet and *S. scombrus* the most specialized one, although these results could be biased because of the poor sample size. *Trachurus trachurus* was already found to be a more specialist predator than *T. mediterraneus* [65]. In the North Adriatic, the largest SEA_C_ is displayed by *S. scombrus*, the species with the highest boreal affinity, while in the South Adriatic *T. trachurus* has the largest SEA_C_, this species being widely spread in these deeper waters. *Trachurus mediterraneus* has the widest trophic niche in the Central Adriatic.

### 4.3. Role of Scomber spp. and Trachurus spp. in the Pelagic Food Web

**Figure 7:**
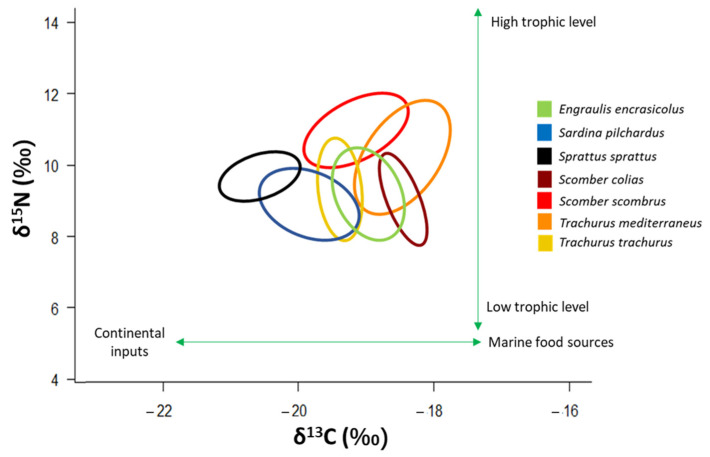
*δ*^13^C-*δ*^15^N scatterplot with standard ellipses corrected for small sample size population (SEA_C,_ ‰^2^) overlaid for the specimens of the four “ancillary” species, and of the three small pelagic species that typify the central part of the pelagic food web in the Adriatic Sea. Signatures of *E. encrasicolus*, *S. pilchardus* and *S. sprattus* are taken from [2].

The four pelagic “ancillary” species studied here showed a trophic position located in the middle of the food web, near other small pelagic species such as *Engraulis encrasicolus*, *Sprattus sprattus* and *Sardina pilchardus* [2]. *Scomber scombrus* and *T. mediterraneus* occupy a slightly higher trophic position than the other small pelagics (Figure 7). The trophic position of these four “ancillary” species confirms their role of pelagic mesopredators that have a trophic niche positioned in the middle of the trophic web [4].

## 5. Conclusions

In conclusion, this study shows that these four pelagic species adopt different strategies to co-exist in the same trophic level and to avoid a complete trophic niche overlap. Intraspecific and spatial resource partitioning is more remarkable in *S. colias* and *T. mediterraneus*, while interspecific resource partitioning is mainly based on different spatial distributions and diet generalism vs. specialism. The results of the SIA confirm those obtained with the SCA but allow the crucial role as mesopredators of these species to be better defined.

Considering their important role and the fact that these species face overexploitation, greater attention should be paid towards monitoring and conserving their stocks.

## Figures and Tables

**Table 1:** Groups and communities considered to run SIBER on SIA results.

	Group	Community
1	*S. colias*, *S. scombrus*, *T. mediterraneus*, *T. trachurus*	1 unique community
2	North, Central and South Adriatic	*S. colias*, *S. scombrus*, *T. mediterraneus*, *T. trachurus*

**Table 2:** Total number of male (M), female (F) and juvenile (ND) specimens of each of the four pelagic species collected at each depth (inshore and offshore position) in each sampling area (North, Central and South Adriatic). Numbers in brackets (x) in the last line indicate the number of empty stomachs found for that species (stomach fullness < 0.5%).

Area	Position	Sex	Species			
			*Scomber colias*	*Scomber scombrus*	*Trachurus mediterraneus*	*Trachurus trachurus*
**North Adriatic**	Inshore	M			32	3
		F		2	37	
		ND		9	3	6
	Offshore	M	5		3	
		F				
		ND			1	
	Total		5 (0)	11 (5)	76 (11)	9 (2)
**Central Adriatic**	Inshore	M			3	
		F		1	8	
		ND	19	4		3
	Offshore	M	2		1	
		F				
		ND	1		1	20
	Total		22 (0)	5 (1)	13 (5)	23 (4)
**South Adriatic**	Inshore	M				
		F				
		ND	13		4	
	Offshore	M	1			
		F	3			
		ND	18			10
	Total		35 (0)	0	4 (0)	10 (2)
**Total males**			8	0	39	3
**Total females**			3	3	45	0
**Total juveniles**			51	13	9	39
**Total individuals**			62 (0)	16 (6)	93 (16)	42 (8)

**Table 3:** Values of Total Area of the convex hull (TA) and of the corrected Standard Ellipse Area (SEA_C_) obtained considering the four different “ancillary” species and the areas in which they were caught. Values of TA and SEA_C_ are expressed as ‰^2^. Other values are shown in the table: mean distance to centroid (CD), *δ*^15^N range (NR), *δ*^13^C range (CR), mean nearest neighbor distance (MNND) and standard deviation of the nearest neighbor distance (SDNND).

SPECIES	*Scomber colias*			*Scomber scombrus*		*Trachurus mediterraneus*			*Trachurus trachurus*		
TA	3.34			5.12		11.3			4.72		
SEAc	1.24			2.26		3.3			1.65		
NR	2.09			0.07		3.28			2.10		
CR	0.35			0.71		0.55			0.33		
CD	0.83			0.36		1.37			0.77		
MNND	0.88			0.71		1.30			1.07		
SDNND	0.63			<0.05		1.31			0.08		
**AREA**	**North**	**Central**	**South**	**North**	**Central**	**North**	**Central**	**South**	**North**	**Central**	**South**
TA	1.09	0.33	1.93	4.64	0.30	5.81	6.17	0.25	0.55	0.88	2.88
SEAc	1.42	0.32	1.21	2.73	0.39	2.21	5.92	0.33	0.45	1.03	1.85

## Data Availability

Data can be requested from the corresponding author upon reasonable request.

## Supplementary Materials

The following supporting information can be downloaded at: https://www.mdpi.com/article/10.3390/biology12020272/s1, Figure S1: Correlation of total length with isotopic signatures; Figure S2: *δ*^13^C-*δ*^15^N scatterplot with standard ellipses corrected for small sample size population (SEA_C,_ ‰^2^) overlaid for the specimens of the four “ancillary” species collected in the North (N), Central (C) and South (S) Adriatic Sea. Table S1: Description of the selected hauls. Table S2: Mean values of total length (cm) and stomach fullness (%). Table S3: Results of univariate PERMANOVA main test carried out on the values of stomach fullness (%) of the four pelagic “ancillary” species. Table S4: List of %W, %N, %F and %IRI values of stomach contents found in *Scomber colias*, in North, Central and South Adriatic Sea. Table S5: Results of multivariate PERMANOVA main test carried out on the diet composition (%W) of each one of the four pelagic “ancillary” species. Table S6: Output of SIMPER analysis conducted on the diet composition (%W) of *S. colias* within each area. Table S7: Results of multivariate PERMANOVA main test carried out on the diet diversity (H’) of the four pelagic “ancillary” species. Table S8: List of %W, %N, %F and %IRI values of stomach contents found in *Scomber scombrus*, in North, Central and South Adriatic Sea. Table S9: Output of SIMPER analysis conducted on the diet composition (%W) of *S. scombrus* within each area. Table S10: List of %W, %N, %F and %IRI values of stomach contents found in *Trachurus mediterraneus*, in North, Central and South Adriatic Sea. Table S11: Output of SIMPER analysis conducted on the diet composition (%W) of *T. mediterraneus* within each area. Table S12: List of %W, %N, %F and %IRI values of stomach contents found in *Trachurus trachurus*, in North, Central and South Adriatic Sea. Table S13: Output of SIMPER analysis conducted on the diet composition (%W) of *T. trachurus* within each area. Table S14: Output of SIMPER analysis conducted on the diet composition (%W) of all the four pelagic “ancillary” species. Table S15: Output of PERMDISP multivariate dispersion test conducted on values of the diet composition (%W) of the four pelagic “ancillary” species. Table S16: Mean values of *δ*^13^C_corrected_ (‰) and *δ*^15^N (‰) measured in the specimens belonging to the four “ancillary” species. Table S17: Results of univariate PERMANOVA main and pairwise tests carried out on the *δ*^13^C, *δ*^15^N and C:N values of the four pelagic “ancillary” species.
