# Supplementary material for: Resource Partitioning among “Ancillary” Pelagic Fishes (Scomber spp., Trachurus spp.) in the Adriatic Sea
Source: Biology (Basel). 2023 Feb 8;12(2):272. doi: 10.3390/biology12020272 (PMC9953290; doi:10.3390/biology12020272)
Supplement: Supplementary file 1 [file biology-12-00272-s001.zip › biology-2147087-supplementary.pdf]

## Supplementary material to:

# Resource partitioning among "ancillary" pelagic fishes (*Scomber* spp., *Trachurus* spp.) in the Adriatic Sea

**Zaira Da Ros[1], Emanuela Fanelli[1,3,*], Sacha Cassatella[1], Ilaria Biagiotti[2], Giovanni Canduci[2], Samuele Menicucci[2], Andrea De Felice[2], Sara Malavolti[2], Iole Leonori[2]**

[1] Department of Life and Environmental Science, Polytechnic University of Marche, 60131 Ancona, Italy; z.daros@pm.univpm.it (ZDR); savino388@gmail.com (SC)

[2] CNR-National Research Council, IRBIM-Institute of Marine Biological Resources and Biotechnologies, Largo Fiera della Pesca, 1, 60125 Ancona, Italy; sara.malavolti@irbim.cnr.it (SMa); iole.leonori@cnr.it (IL); ilaria.biagiotti@cnr.it (IB); giovanni.canduci@cnr.it (GC); samuele.menicucci@irbim.cnr.it (SMe); andrea.defelice@cnr.it (ADF)

* Correspondence: e.fanelli@univpm.it

**Supplementary Table S1.** Description of the selected hauls.

| Haul number | Date | Starting time | Subarea | Depth (m) | Position | Day/Night |
|---|---|---|---|---|---|---|
| 8 | 12/06/2019 | 10:14:00 | Northern Adriatic | 25 | Inshore | Day |
| 9 | 13/06/2019 | 09:54:00 | Northern Adriatic | 26 | Inshore | Day |
| 11 | 14/06/2019 | 08:04:00 | Northern Adriatic | 30 | Inshore | Day |
| 12 | 14/06/2019 | 16:30:00 | Northern Adriatic | 24 | Inshore | Day |
| 16 | 15/06/2019 | 21:33:00 | Northern Adriatic | 42 | Offshore | Night |
| 17 | 16/06/2019 | 08:28:00 | Northern Adriatic | 22 | Inshore | Day |
| 19 | 18/06/2019 | 06:41:00 | Northern Adriatic | 20 | Inshore | Day |
| 20 | 18/06/2019 | 15:59:00 | Northern Adriatic | 19 | Inshore | Day |
| 21 | 18/06/2019 | 21:45:00 | Northern Adriatic | 65 | Offshore | Night |
| 24 | 26/06/2019 | 15:43:00 | Northern Adriatic | 23 | Inshore | Day |
| 25 | 26/06/2019 | 21:40:00 | Northern Adriatic | 76 | Offshore | Night |
| 28 | 28/06/2019 | 10:30:00 | Northern Adriatic | 28 | Inshore | Day |
| 30 | 01/07/2019 | 21:42:00 | Central Adriatic | 163 | Offshore | Night |
| 31 | 02/07/2019 | 07:38:00 | Central Adriatic | 22 | Inshore | Day |
| 33 | 03/07/2019 | 07:38:00 | Central Adriatic | 28 | Inshore | Day |
| 34 | 03/07/2019 | 21:39:00 | Central Adriatic | 119 | Offshore | Night |
| 37 | 06/07/2019 | 21:38:00 | Central Adriatic | 109 | Offshore | Night |
| 38 | 07/07/2019 | 09:42:00 | Central Adriatic | 22 | Inshore | Day |
| 40 | 07/07/2019 | 21:50:00 | Southern Adriatic | 151 | Offshore | Night |
| 41 | 08/07/2019 | 16:27:00 | Southern Adriatic | 40 | Inshore | Day |
| 42 | 08/07/2019 | 21:37:00 | Southern Adriatic | 188 | Offshore | Night |
| 43 | 09/07/2019 | 08:38:00 | Southern Adriatic | 38 | Inshore | Day |
| 46 | 15/07/2019 | 21:36:00 | Southern Adriatic | 120 | Offshore | Night |

**Supplementary Table S2.** Mean values of stomach fullness (%) measured in the specimens belonging to the four "ancillary" species caught in the North, Central, and South Adriatic Sea. S.D. = standard deviation.

| | | **Stomach fullness (%)** | |
|---|---|---|---|
| **Species** | **Area** | mean | S.D. |
| *Scomber colias* | North | 2.14 | 1.20 |
| | Central | 3.19 | 2.53 |
| | South | 4.78 | 5.15 |
| *Scomber scombrus* | North | 0.17 | 0.17 |
| | Central | 1.05 | 1.48 |
| *Trachurus mediterraneus* | North | 0.31 | 5.34 |
| | Central | 0.87 | 1.52 |
| | South | 3.67 | 0.73 |
| *Trachurus trachurus* | North | 0.34 | 0.51 |
| | Central | 2.70 | 1.56 |
| | South | 2.01 | 1.67 |

**Supplementary Table S3.** Results of univariate PERMANOVA main test carried out on the values of stomach fullness (%) of the four "ancillary" pelagic species. The factors used was the factor "Area" with three levels (North, Central and South Adriatic). df=degrees of freedom; MS= mean square; Pseudo-F= statistic F; P(MC)= probability level after Monte Carlo test; *=p ≤ 0.05; **=p ≤ 0.01; ***=p ≤ 0.001; n.s. = not significant.

| PERMANOVA main test on STOMACH FULLNESS | | | | | |
|---|---|---|---|---|---|
| **Species** | **Source** | **df** | **MS** | **Pseudo-F** | **P(MC)** |
| *Scomber colias* | Area | 2 | 28 | 1.91 | n.s. |
| | Residuals | 59 | 14.46 | | |
| | Total | 61 | | | |
| **Species** | **Source** | **df** | **MS** | **Pseudo-F** | **P(MC)** |
| *Scomber scombrus* | Area | 1 | 2.65 | 4.09 | n.s. |
| | Residuals | 14 | 0.65 | | |
| | Total | 15 | | | |
| **Species** | **Source** | **df** | **MS** | **Pseudo-F** | **P(MC)** |
| *Trachurus mediterraneus* | Area | 2 | 21.79 | 47.88 | ***. |
| | Residuals | 74 | 0.46 | | |
| | Total | 76 | | | |
| **Species** | **Source** | **df** | **MS** | **Pseudo-F** | **P(MC)** |
| *Trachurus trachurus* | Area | 2 | 9.75 | 4.23 | * |
| | Residuals | 31 | 2.3 | | |
| | Total | 33 | | | |
| **Species** | **Source** | **df** | **MS** | **Pseudo-F** | **P(MC)** |
| All species | Species | 3 | 25.27 | 1.86 | n.s. |
| | Area(Species) | 7 | 17.39 | 2.69 | *** |
| | Residuals | 178 | 6.47 | | |
| | Total | 188 | | | |

**Supplementary Table S4.** List of %W, %N, %F and %IRI values of stomach contents found in *Scomber colias,* in North, Central and South Adriatic Sea.

| | North Adriatic | | | | | Central Adriatic | | | | | South Adriatic | | | | |
|---|---|---|---|---|---|---|---|---|---|---|---|---|---|---|---|
| | **% W** | **% N** | **% F** | **IRI** | **% IRI** | **% W** | **% N** | **% F** | **IRI** | **% IRI** | **% W** | **% N** | **% F** | **IRI** | **% IRI** |
| **CNIDARIA** | | | | | | | | | | | | | | | |
| Calycophorae | - | - | - | - | - | 3.28 | 2.49 | 40.91 | 236.15 | 2.99 | 1.06 | 0.47 | 22.86 | 35.02 | 0.31 |
| **Total Cnidaria** | **-** | **-** | **-** | **-** | **-** | **3.28** | **2.49** | **40.91** | **236.15** | **2.99** | **1.06** | **0.47** | **22.86** | **35.02** | **0.31** |
| **MOLLUSCA** | | | | | | | | | | | | | | | |
| *Creseis acicula* | - | - | - | - | - | - | - | - | - | - | 0.02 | 0.18 | 17.14 | 3.44 | 0.03 |
| Sepiolidae | 3.39 | 0.09 | 20.00 | 69.65 | 0.48 | - | - | - | - | - | 10.80 | 0.56 | 20.00 | 227.25 | 2.02 |
| Bivalvia larvae | - | - | - | - | - | - | - | - | - | - | 0.18 | 0.35 | 11.43 | 6.14 | 0.05 |
| **Total Mollusca** | **3.39** | **0.09** | **20.00** | **69.65** | **0.48** | **-** | **-** | **-** | **-** | **-** | **11.01** | **1.09** | **48.57** | **236.82** | **2.10** |
| **CRUSTACEA** | | | | | | | | | | | | | | | |
| **Copepoda** | | | | | | | | | | | | | | | |
| *Calanus*-like | 0.00 | 0.09 | 20.00 | 1.84 | 0.01 | 0.01 | 0.01 | 0.25 | 9.09 | 0.12 | 0.00 | 0.03 | 2.86 | 0.08 | 0.00 |
| *Centropages* sp. | 0.03 | 1.53 | 60.00 | 93.67 | 0.64 | 0.04 | 0.04 | 1.50 | 22.73 | 0.29 | 0.00 | 0.15 | 2.86 | 0.43 | 0.00 |
| *Euchaeta* sp. | 0.01 | 0.09 | 20.00 | 1.94 | 0.01 | 0.07 | 1.00 | 18.18 | 19.33 | 0.24 | 0.03 | 0.47 | 28.57 | 14.22 | 0.13 |
| *Oncaea* sp. | 0.00 | 0.09 | 20.00 | 1.87 | 0.01 | - | - | - | - | - | 0.00 | 0.03 | 2.86 | 0.08 | 0.00 |
| *Sapphirina* sp. | - | - | - | - | - | - | - | - | - | - | 0.01 | 0.32 | 22.86 | 7.66 | 0.07 |
| *Temora stylifera* | - | - | - | - | - | - | - | - | - | - | 0.00 | 0.03 | 2.86 | 0.09 | 0.00 |
| Unid. Copepoda | 0.00 | 0.09 | 20.00 | 1.84 | 0.01 | 0.02 | 1.00 | 22.73 | 23.11 | 0.29 | 0.01 | 0.47 | 37.14 | 17.68 | 0.16 |
| **Total Copepoda** | **0.04** | **1.89** | **140.00** | **101.15** | **0.69** | **0.13** | **2.04** | **42.65** | **74.25** | **0.94** | **0.05** | **1.50** | **100.00** | **40.25** | **0.36** |
| **Stomatopoda** | | | | | | | | | | | | | | | |
| Squillidae larvae | - | - | - | - | - | 0.25 | 0.12 | 4.55 | 1.69 | 0.02 | 1.36 | 0.94 | 31.43 | 72.36 | 0.64 |
| **Total Stomatopoda** | **-** | **-** | **-** | **-** | **-** | **0.25** | **0.12** | **4.55** | **1.69** | **0.02** | **1.36** | **0.94** | **31.43** | **72.36** | **0.64** |
| **Euphausiacea** | | | | | | | | | | | | | | | |
| *Nematoscelis megalops* | - | - | - | - | - | - | - | - | - | - | 0.86 | 0.50 | 8.57 | 11.65 | 0.10 |
| *Nyctiphanes couchii* | - | - | - | - | - | - | - | - | - | - | 18.86 | 11.15 | 28.57 | 857.47 | 7.61 |
| Euphausiidae larvae | - | - | - | - | - | - | - | - | - | - | 1.05 | 7.13 | 20.00 | 163.74 | 1.45 |
| Euphausiidae | - | - | - | - | - | - | - | - | - | - | 0.20 | 0.23 | 8.57 | 3.72 | 0.03 |
| **Total Euphausiacea** | **-** | **-** | **-** | **-** | **-** | **-** | **-** | **-** | **-** | **-** | **20.97** | **19.02** | **65.71** | **1036.58** | **9.20** |
| **Decapoda** | | | | | | | | | | | | | | | |

| | | | | | | | | | | | | | | | |
|---|---|---|---|---|---|---|---|---|---|---|---|---|---|---|---|
| Megalopa Brachyura | - | - | - | - | - | 0.05 | 1.00 | 9.09 | 9.57 | 0.12 | 1.17 | 3.14 | 31.43 | 135.54 | 1.20 |
| Zoea Brachyura | - | - | - | - | - | - | - | - | - | - | 0.05 | 0.21 | 8.57 | 2.17 | 0.02 |
| Zoea Penaeidae | - | - | - | - | - | 0.58 | 0.12 | 4.55 | 3.19 | 0.04 | 0.03 | 0.12 | 2.86 | 0.41 | 0.00 |
| Zoea Palaemonidae | - | - | - | - | - | - | - | - | - | - | 0.02 | 0.09 | 8.57 | 0.97 | 0.01 |
| **Total Decapoda** | **-** | **-** | **-** | **-** | **-** | **0.63** | **1.12** | **13.64** | **12.75** | **0.16** | **1.27** | **3.55** | **51.43** | **139.09** | **1.23** |
| **Amphipoda** | | | | | | | | | | | | | | | |
| *Anchylomera blossevillei* | - | - | - | - | - | - | - | - | - | - | 0.14 | 0.23 | 14.29 | 5.41 | 0.05 |
| *Brachyscelus crusculum* | - | - | - | - | - | - | - | - | - | - | 0.05 | 0.03 | 2.86 | 0.24 | 0.00 |
| *Lestrigonus schizogeneios* | - | - | - | - | - | - | - | - | - | - | 0.07 | 0.26 | 2.86 | 0.94 | 0.01 |
| *Phronima atlantica* | 1.51 | 0.54 | 80.00 | 163.75 | 1.12 | 1.75 | 0.75 | 9.09 | 22.71 | 0.29 | 0.41 | 0.50 | 37.14 | 33.91 | 0.30 |
| *Phronima stebbingi* | - | - | - | - | - | - | - | - | - | - | 0.02 | 0.03 | 2.86 | 0.14 | 0.00 |
| *Phronimella elongata* | - | - | - | - | - | - | - | - | - | - | 0.01 | 0.06 | 5.71 | 0.37 | 0.00 |
| *Phrosina semilunata* | - | - | - | - | - | - | - | - | - | - | 0.32 | 0.26 | 11.43 | 6.66 | 0.06 |
| *Platyscelus* sp. | - | - | - | - | - | - | - | - | - | - | 0.01 | 0.03 | 2.86 | 0.11 | 0.00 |
| *Primno macropa* | - | - | - | - | - | - | - | - | - | - | 0.03 | 0.06 | 5.71 | 0.50 | 0.00 |
| *Themisto abyssorum* | - | - | - | - | - | 0.05 | 0.62 | 13.64 | 9.21 | 0.12 | 2.11 | 4.43 | 42.86 | 280.47 | 2.49 |
| *Vibilia* sp. | 0.00 | 0.09 | 20.00 | - | - | - | - | - | - | - | 0.07 | 0.15 | 11.43 | 2.53 | 0.02 |
| Hyperiidae | 0.73 | 8.75 | 80.00 | 757.97 | 5.20 | 0.74 | 0.74 | 3.37 | 22.73 | 0.29 | 0.02 | 1.44 | 17.14 | 25.07 | 0.22 |
| Hyperiidea | 0.00 | 0.09 | 20.00 | - | - | - | - | - | - | - | 0.02 | 0.53 | 14.29 | 7.88 | 0.07 |
| **Total Amphipoda** | **2.24** | **9.47** | **200.00** | **921.72** | **6.32** | **2.55** | **2.12** | **26.09** | **54.65** | **0.69** | **3.29** | **8.01** | **171.43** | **364.22** | **3.23** |
| **Total Crustacea** | **2.28** | **11.36** | **340.00** | **1022.87** | **7.02** | **3.56** | **5.40** | **86.93** | **143.34** | **1.82** | **5.98** | **14.00** | **354.29** | **615.91** | **5.47** |
| **THALIACEA** | | | | | | | | | | | | | | | |
| *Pyrosoma* sp. | - | - | - | - | - | - | - | - | - | - | 1.66 | 0.06 | 5.71 | 9.85 | 0.09 |
| Salpidae | 44.73 | 82.15 | 80.00 | 10150.45 | 69.64 | 37.43 | 51.87 | 54.55 | 4871.15 | 61.74 | 24.45 | 58.17 | 100.00 | 8262.34 | 73.35 |
| **Total Thaliacea** | **44.73** | **82.15** | **80.00** | **10150.45** | **69.64** | **37.43** | **51.87** | **54.55** | **4871.15** | **61.74** | **26.11** | **58.23** | **105.71** | **8272.18** | **73.44** |
| **OSTEICHTHYES** | | | | | | | | | | | | | | | |
| *Conger conger* | - | - | - | - | - | - | - | - | - | - | 0.51 | 0.03 | 2.86 | 1.54 | 0.01 |
| *Engraulis encrasicolus* juvenile | - | - | - | - | - | - | - | - | - | - | 3.10 | 0.12 | 11.43 | 36.78 | 0.33 |
| *E. encrasic.* larvae | - | - | - | - | - | 4.19 | 1.12 | 9.09 | 48.29 | 0.61 | 23.87 | 4.08 | 31.43 | 878.53 | 7.80 |
| *Serranus* sp. larvae | - | - | - | - | - | 1.14 | 0.12 | 4.55 | 5.74 | 0.07 | 2.39 | 0.59 | 17.14 | 50.97 | 0.45 |
| *Scomber* sp. | - | - | - | - | - | - | - | - | - | - | 1.14 | 0.03 | 2.86 | 3.33 | 0.03 |
| *Scomber* sp. larvae | - | - | - | - | - | - | - | - | - | - | 0.82 | 0.15 | 5.71 | 5.53 | 0.05 |
| *Trachurus mediterraneus* | - | - | - | - | - | - | - | - | - | - | 1.85 | 0.03 | 2.86 | 5.37 | 0.05 |

| | | | | | | | | | | | | | | | |
|---|---|---|---|---|---|---|---|---|---|---|---|---|---|---|---|
| *Uranoscopus scaber* | 0.66 | 0.09 | 20.00 | 14.93 | 0.10 | - | - | - | - | - | - | - | - | - | - |
| Fish skeleton | 2.46 | 0.63 | 20.00 | 61.85 | 0.42 | 39.72 | 4.61 | 50.00 | 2216.72 | 28.10 | 0.95 | 0.76 | 20.00 | 34.29 | 0.30 |
| **Total Osteichthyes** | **3.12** | **0.72** | **40.00** | **76.79** | **0.53** | **45.05** | **5.86** | **63.64** | **2270.74** | **28.78** | **34.63** | **5.78** | **94.29** | **1016.35** | **9.02** |
| **OTHERS** | | | | | | | | | | | | | | | |
| Unid.digestive pulp | 44.91 | 0.27 | 60.00 | 2710.88 | 18.60 | - | - | - | - | - | - | - | - | - | - |
| Scales | 1.45 | 4.87 | 80.00 | 505.35 | 3.47 | 10.68 | 30.05 | 9.09 | 370.31 | 4.69 | 0.08 | 0.08 | 0.59 | 20.00 | 0.18 |
| *Anisakis* sp. | - | - | - | - | - | - | - | - | - | - | 0.07 | 0.07 | 0.06 | 5.71 | 0.05 |
| Trematoda parasites | 0.11 | 0.54 | 60.00 | 39.24 | 0.27 | - | - | - | - | - | 0.08 | 0.08 | 0.76 | 25.71 | 0.23 |
| **Total Others** | **46.47** | **5.68** | **200.00** | **3255.47** | **22.34** | **10.68** | **30.05** | **9.09** | **370.31** | **4.69** | **0.22** | **0.22** | **1.41** | **51.43** | **0.46** |

**Supplementary Table S5.** Results of multivariate PERMANOVA main test carried out on the diet composition (%W) of each one of the four "ancillary" pelagic species. The factor used was factor "Area" with three levels (North, Central and South Adriatic). df=degrees of freedom; MS= mean square; Pseudo-F= statistic F; t=statistic t for pairwise comparisons; Unique perms= number of permutations; P(MC)= probability level after Monte Carlo test; *=p < 0.05; **=p < 0.01; ***=p < 0.001; n.s. = not significant.

| PERMANOVA main test on diet composition (%W) | | | | | |
|---|---|---|---|---|---|
| **Species** | **Source** | **df** | **MS** | **Pseudo-F** | **P(MC)** |
| *Scomber colias* | Area | 2 | 275.48 | 3.26 | * |
| | Residuals | 59 | 963.15 | | |
| | Total | 61 | | | |
| **Species** | **Source** | **df** | **MS** | **Pseudo-F** | **P(MC)** |
| *Scomber scombrus* | Area | 1 | 16.87 | 1.06 | n.s. |
| | Residuals | 14 | 15.85 | | |
| | Total | 15 | | | |
| **Species** | **Source** | **df** | **MS** | **Pseudo-F** | **P(MC)** |
| *Trachurus mediterraneus* | Area | 2 | 6.63 | 0.92 | n.s. |
| | Residuals | 74 | 7.18 | | |
| | Total | 76 | | | |
| **Species** | **Source** | **df** | **MS** | **Pseudo-F** | **P(MC)** |
| *Trachurus trachurus* | Area | 2 | 35.17 | 6.15 | ** |
| | Residuals | 31 | 5.72 | | |
| | Total | 33 | | | |
| **Species** | **Source** | **df** | **MS** | **Pseudo-F** | **P(MC)** |
| All species | Species | 3 | 139.66 | 2 | n.s. |
| | Area(Species) | 7 | 93.06 | 3 | *** |
| | Residuals | 188 | | | |

**Supplementary Table S6.** Output of SIMPER analysis conducted on the diet composition (%W) of *S. colias* within each Area (N= North Adriatic, C= Central Adriatic, S= South Adriatic). SIMPER analysis was conducted on the Bray Curtis resemblance matrix of transformed biomass data. Only comparisons between neighbouring sub-areas are shown. Cut-off for low contribution at 60%. Av.Abund = average abundance; Av. Sim = average similarity; Contrib %= percentage of variance explained by the explanatory variables; Cum. %= cumulative percentage of variance explained by the explanatory variables.

| | | | | |
|---|---|---|---|---|
| | | **Group N** | | |
| | | **Average similarity: 24.87 %** | | |
| Species | Av.Abund | Av.Sim | Contrib% | Cum.% |
| *P.atlantica* | 0.01 | 13.04 | 52.43 | 52.43 |
| Salpidae spp. | 0.09 | 11.28 | 45.34 | 97.77 |
| | | **Group C** | | |
| | | **Average similarity: 16.64 %** | | |
| Species | Av.Abund | Av.Sim | Contrib% | Cum.% |
| Fish skeleton | 0.03 | 10.34 | 62.14 | 62.14 |
| | | **Group S** | | |
| | | **Average similarity: 28.18** | | |
| Species | Av.Abund | Av.Sim | Contrib% | Cum.% |
| Salpidae | 0.06 | 24.80 | 88.00 | 88.00 |
| | | **Groups N & C** | | |
| | | **Average dissimilarity = 92.35 %** | | |
| | Group N | Group C | | |
| Species | Av.Abund | Av.Abund | Contrib% | Cum.% |
| Salpidae | 0.09 | 0.03 | 50.56 | 50.56 |
| Fish skeleton | 0.00 | 0.03 | 21.06 | 71.62 |
| | | **Groups N & S** | | |
| | | **Average dissimilarity = 83.35 %** | | |
| | Group N | Group S | | |
| Species | Av.Abund | Av.Abund | Contrib% | Cum.% |
| Salpidae | 0.09 | 0.06 | 51.50 | 51.50 |
| *Phronima atlantica* | 0.01 | 0.00 | 12.64 | 64.14 |
| | | **Groups C & S** | | |
| | | **Average dissimilarity = 87,23 %** | | |
| | Group C | Group S | | |
| Species | Av.Abund | Av.Abund | Contrib% | Cum.% |
| Salpidae | 0.03 | 0.06 | 39.83 | 39.83 |
| Fish skeleton | 0.03 | 0.00 | 18.22 | 58.05 |
| *Engraulis encrasicolus* larvae | 0.00 | 0.05 | 9.72 | 67.77 |

**Supplementary Table S7.** Results of multivariate PERMANOVA main test carried out on the diet diversity (H') of the four "ancillary" pelagic species. The factors used was the factor "Area" with three levels (North, Central and South Adriatic). df=degrees of freedom; MS= mean square; Pseudo-F= statistic F; P(MC)= probability level after Monte Carlo test; *=p ≤ 0.05; **=p ≤ 0.01; ***=p ≤ 0.001; n.s. = not significant.

| **PERMANOVA main test on DIET DIVERSITY (H')** | | | | | |
|---|---|---|---|---|---|
| **Species** | **Source** | **df** | **MS** | **Pseudo-F** | **P(MC)** |
| *Scomber colias* | Area | 2 | 1.77 | 5.73 | ** |
| | Residuals | 59 | 0.31 | | |
| | Total | 61 | | | |
| **Species** | **Source** | **df** | **MS** | **Pseudo-F** | **P(MC)** |
| *Scomber scombrus* | Area | 1 | 0.20 | 1.38 | n.s. |
| | Residuals | 14 | 0.15 | | |
| | Total | 15 | | | |
| **Species** | **Source** | **df** | **MS** | **Pseudo-F** | **P(MC)** |
| *Trachurus mediterraneus* | Area | 2 | 0.48 | 0.64 | n.s. |
| | Residuals | 71 | 0.18 | | |
| | Total | 73 | | | |
| **Species** | **Source** | **df** | **MS** | **Pseudo-F** | **P(MC)** |
| *Trachurus trachurus* | Area | 2 | 1.22 | 8.62 | ** |
| | Residuals | 31 | 0.14 | | |
| | Total | 33 | | | |
| **Species** | **Source** | **df** | **MS** | **Pseudo-F** | **P(MC)** |
| All species | Species | 3 | 0.22 | 0.30 | n.s. |
| | Area(Species) | 7 | 1.02 | 4.74 | *** |
| | Residuals | 175 | 022 | | |
| | Total | 185 | | | |

**Supplementary Table S8.** List of %W, %N, %F and %IRI values of stomach contents found in *Scomber scombrus,* in North and Central Adriatic Sea.

| | North Adriatic | | | | | Central Adriatic | | | | |
|---|---|---|---|---|---|---|---|---|---|---|
| | **W%** | **N%** | **F%** | **IRI** | **IRI%** | **W%** | **N%** | **F%** | **IRI** | **IRI%** |
| **MOLLUSCA** | | | | | | | | | | |
| Sepiolidae | - | - | - | - | - | 1.06 | 0.67 | 20.00 | 34.54 | 0.35 |
| **Total Mollusca** | **-** | **-** | **-** | **-** | **-** | **1.06** | **0.67** | **20.00** | **34.54** | **0.35** |
| **CRUSTACEA** | | | | | | | | | | |
| **Decapoda** | | | | | | | | | | |
| Zoea Galatheidae | - | - | - | - | - | 0.79 | 1.33 | 20.00 | 42.39 | 0.43 |
| **Total Decapoda** | **-** | **-** | **-** | **-** | **-** | **0.79** | **1.33** | **20.00** | **42.39** | **0.43** |
| **Amphipoda** | | | | | | | | | | |
| *Anchylomera blossevillei* | - | - | - | - | - | 0.71 | 0.67 | 20.00 | 27.50 | 0.28 |
| Hyperiidea | - | - | - | - | - | 0.23 | 0.67 | 20.00 | 17.90 | 0.18 |
| **Total Amphipoda** | **-** | **-** | **-** | **-** | **-** | **0.94** | **1.33** | **40.00** | **45.40** | **0.46** |
| **Total Crustacea** | **-** | **-** | **-** | **-** | **-** | **1.72** | **2.67** | **60.00** | **87.79** | **0.89** |
| **THALIACEA** | | | | | | | | | | |
| *Pyrosoma* sp. | 37.74 | 3.33 | 18.18 | 746.79 | 5.67 | - | - | - | - | - |
| Salpidae | - | - | - | - | - | 1.70 | 8.00 | 20.00 | 194.00 | 1.97 |
| **Total Thaliacea** | **37.74** | **3.33** | **18.18** | **746.79** | **5.67** | **1.70** | **8.00** | **20.00** | **194.00** | **1.97** |
| **OSTEICHTHYES** | | | | | | | | | | |
| Clupeiformes | - | - | - | - | - | 75.73 | 0.67 | 20.00 | 1528.00 | 15.50 |
| Fish skeleton | - | - | - | - | - | 9.43 | 0.67 | 20.00 | 201.98 | 2.05 |
| **Total Osteichthyes** | **-** | **-** | **-** | **-** | **-** | **85.17** | **1.33** | **40.00** | **1729.98** | **17.54** |
| **OTHERS** | | | | | | | | | | |
| Scales | 26.28 | 38.33 | 72.73 | 4699.30 | 35.70 | 9.56 | 77.33 | 80.00 | 6951.13 | 70.49 |
| Trematoda parasites | 35.98 | 58.33 | 81.82 | 7716.35 | 58.62 | 0.80 | 10 | 80 | 863.61 | 8.76 |
| **Total Others** | **62.26** | **96.67** | **154.55** | **12415.65** | **94.33** | **10.35** | **87.33** | **160.00** | **7814.74** | **79.25** |

**Supplementary Table S9.** Output of SIMPER analysis conducted on the diet composition (%W) of *S. scombrus* within each Area (N= North Adriatic, C= Central Adriatic, S= South Adriatic). SIMPER analysis was conducted on the Bray Curtis resemblance matrix of transformed biomass data. Only comparisons between neighbouring sub-areas are shown. Cut-off for low contribution at 60%. Av.Abund = average abundance; Av. Sim = average similarity; Contrib %= percentage of variance explained by the explanatory variables; Cum. %= cumulative percentage of variance explained by the explanatory variables.

| | | **Group N** | | |
|---|---|---|---|---|
| | | **Average similarity: 0.64 %** | | |
| Species | Av.Abund | Av.Sim | Contrib% | Cum.% |
| *Pyrosoma* sp. | 0.00 | 0.64 | 100.00 | 100.00 |
| | | **Group C** | | |
| | | **Average similarity: - %** | | |

| | | **Groups N & C** | | |
|---|---|---|---|---|
| | | **Average dissimilarity = 100 %** | | |
| | **Group N** | **Group C** | | |
| Species | Av.Abund | Av.Abund | Contrib% | Cum.% |
| Clupeiforme | 0.00 | 0.07 | 23.80 | 23.80 |
| Fish skeleton | 0.00 | 0.02 | 23.46 | 47.26 |
| Sepiolidae | 0 | 0 | 22.26 | 69.51 |

**Supplementary Table S10.** List of %W, %N, %F and %IRI values of stomach contents found in *Trachurus mediterraeus,* in North, Central and South Adriatic Sea.

| | North Adriatic | | | | | Central Adriatic | | | | | South Adriatic | | | | |
|---|---|---|---|---|---|---|---|---|---|---|---|---|---|---|---|
| | **W%** | **N%** | **F%** | **IRI** | **IRI%** | **W%** | **N%** | **F%** | **IRI** | **IRI%** | **W%** | **N%** | **F%** | **IRI** | **IRI%** |
| **MOLLUSCA** | | | | | | | | | | | | | | | |
| *Creseis acicula* | 0.00 | 0.01 | 1.67 | 0.03 | 0.00 | 0.00 | 1.16 | 7.69 | 8.94 | 0.23 | 0.18811 | 2.76498 | 25 | 73.83 | 0.69 |
| Gastropoda unid. | 0.26 | 0.18 | 10.00 | 4.41 | 0.12 | 0.01 | 0.58 | 7.69 | 4.55 | 0.12 | - | - | - | - | - |
| Bivalvia unid. | 0.51 | 0.35 | 18.33 | 15.80 | 0.43 | 0.01 | 1.16 | 7.69 | 9.05 | 0.24 | - | - | - | - | - |
| *Illex coindetii* | - | - | - | - | - | 71.95 | 0.58 | 7.69 | 557.96 | 14.53 | - | - | - | - | - |
| **Total Mollusca** | **0.77** | **0.55** | **30.00** | **20.24** | **0.55** | **71.98** | **3.49** | **30.77** | **580.51** | **15.12** | **0.19** | **2.76** | **25.00** | **73.83** | **0.69** |
| **CRUSTACEA** | | | | | | | | | | | | | | | |
| **Cladocera** | | | | | | | | | | | | | | | |
| Unid. Cladocera | 0.00 | 0.01 | 1.67 | 0.03 | 0.00 | - | - | - | - | - | 0.07 | 0.46 | 25.00 | 13.33 | 0.12 |
| **Total Cladocera** | **0.00** | **0.01** | **1.67** | **0.03** | **0.00** | - | - | - | - | - | **0.07** | **0.46** | **25.00** | **13.33** | **0.12** |
| **Copepoda** | | | | | | | | | | | | | | | |
| *Acartia* sp. | 5.39 | 64.50 | 23.33 | 1630.73 | 44.60 | - | - | - | - | - | - | - | - | - | - |
| *Aetideus* sp. | 0.01 | 0.04 | 5.00 | 0.24 | 0.01 | - | - | - | - | - | - | - | - | - | - |
| *Calanus*-like | 0.11 | 0.31 | 3.33 | 1.40 | 0.04 | - | - | - | - | - | 10.35 | 40.55 | 100.00 | 5089.88 | 42.64 |
| *Centropages* sp. | - | - | - | - | - | - | - | - | - | - | 0.37 | 3.23 | 100.00 | 359.48 | 3.35 |
| *Euchaeta* sp. | - | - | - | - | - | 0.21 | 7.56 | 7.69 | 59.76 | 1.56 | 11.14 | 13.36 | 50.00 | 1225.29 | 10.82 |
| *Euterpina acutifrons* | 0.03 | 0.25 | 8.33 | 2.30 | 0.06 | - | - | - | - | - | - | - | - | - | - |
| *Oncaea mediteranea* | - | - | - | - | - | - | - | - | - | - | 0.07 | 0.46 | 25.00 | 13.33 | 0.08 |
| *Sapphirina* sp. | - | - | - | - | - | - | - | - | - | - | 0.33 | 0.46 | 25.00 | 19.66 | 0.11 |
| *Temora stylifera* | 0.01 | 0.05 | 5.00 | 0.33 | 0.01 | 0.00 | 0.58 | 7.69 | 4.47 | 0.12 | - | - | - | - | - |
| **Total Copepoda** | **5.55** | **65.15** | **45.00** | **1635.01** | **44.71** | **0.21** | **8.14** | **15.38** | **64.23** | **1.67** | **22.25** | **58.06** | **300.00** | **6707.64** | **57.01** |
| **Ostracoda** | | | | | | | | | | | | | | | |
| Unid. Ostracoda | 0.08 | 0.38 | 16.67 | 7.59 | 0.21 | - | - | - | - | - | - | - | - | - | - |
| **Total Ostracoda** | **0.08** | **0.38** | **16.67** | **7.59** | **0.21** | **0.00** | **0.00** | **0.00** | **0.00** | **0.00** | - | - | - | - | - |
| **Stomatopoda** | | | | | | | | | | | | | | | |
| Squillidae larvae | - | - | - | - | - | - | - | - | - | - | 21.20 | 1.84 | 25.00 | 576.04 | 5.33 |
| **Total Stomatopoda** | - | - | - | - | - | - | - | - | - | - | **21.20** | **1.84** | **25.00** | **576.04** | **5.33** |
| **Euphausiacea** | | | | | | | | | | | | | | | |
| Euphausiidae larvae | - | - | - | - | - | - | - | - | - | - | 1.59 | 2.30 | 25.00 | 97.40 | 0.91 |

| | | | | | | | | | | | | | | | |
|---|---|---|---|---|---|---|---|---|---|---|---|---|---|---|---|
| **Total Euphausiacea** | **-** | **-** | **-** | **-** | **-** | **-** | **-** | **-** | **-** | **-** | **1.59** | **2.30** | **25.00** | **97.40** | **0.91** |
| **Decapoda** | | | | | | | | | | | | | | | |
| *Alpheus glaber* | 10.60 | 0.04 | 3.33 | 35.45 | 0.97 | 12.07 | 1.16 | 15.38 | 203.51 | 5.30 | - | - | - | - | - |
| *Philocheras bispinosus* | 1.57 | 0.13 | 8.33 | 14.15 | 0.39 | - | - | - | - | - | - | - | - | - | - |
| *Liocarcinus depurator* | 14.90 | 0.01 | 1.67 | 24.86 | 0.68 | - | - | - | - | - | - | - | - | - | - |
| *Processa* sp. | - | - | - | - | - | 4.85 | 2.33 | 7.69 | 55.23 | 1.44 | - | - | - | - | - |
| Penaeidae | 10.85 | 0.04 | 5.00 | 54.47 | 1.49 | - | - | - | - | - | - | - | - | - | - |
| Brachyura | 0.94 | 0.04 | 3.33 | 3.25 | 0.09 | - | - | - | - | - | - | - | - | - | - |
| Megalopa Brachyura | 0.26 | 0.16 | 6.67 | 2.80 | 0.08 | 0.00 | 1.16 | 15.38 | 17.96 | 0.47 | - | - | - | - | - |
| Zoea Brachyura | 0.01 | 0.05 | 3.33 | 0.19 | 0.01 | - | - | - | - | - | - | - | - | - | - |
| Zoea Caridea | - | - | - | - | - | - | - | - | - | - | 18.74 | 20.28 | 50.00 | 1950.74 | 18.21 |
| **Total Decapoda** | **39.12** | **0.47** | **31.67** | **135.18** | **3.70** | **16.92** | **4.65** | **38.46** | **276.70** | **7.21** | **18.74** | **20.28** | **50.00** | **1950.74** | **18.21** |
| **Mysida** | | | | | | | | | | | | | | | |
| *Siriella* sp. | 17.25 | 4.09 | 15.00 | 320.15 | 8.76 | 1.04 | 2.91 | 7.69 | 30.34 | 0.79 | - | - | - | - | - |
| Mysidae | 0.20 | 0.05 | 1.67 | 0.42 | 0.01 | 0.48 | 1.74 | 15.38 | 34.26 | 0.89 | - | - | - | - | - |
| **Total Mysida** | **17.45** | **4.15** | **16.67** | **320.57** | **8.77** | **1.52** | **4.65** | **23.08** | **64.61** | **1.68** | **-** | **-** | **-** | **-** | **-** |
| **Cumacea** | | | | | | | | | | | | | | | |
| *Cumella pigmea* | 0.02 | 0.03 | 3.33 | 0.14 | 0.00 | - | - | - | - | - | - | - | - | - | - |
| *Iphinoe* sp. | 0.03 | 0.04 | 3.33 | 0.23 | 0.01 | - | - | - | - | - | - | - | - | - | - |
| **Total Cumacea** | **0.04** | **0.07** | **6.67** | **0.37** | **0.01** | **-** | **-** | **-** | **-** | **-** | **-** | **-** | **-** | **-** | **-** |
| **Isopoda** | | | | | | | | | | | | | | | |
| *Anilocra frontalis* | 0.73 | 0.01 | 1.67 | 1.23 | 0.03 | - | - | - | - | - | - | - | - | - | - |
| *Sphaeroma* sp. | 0.04 | 0.03 | 1.67 | 0.12 | 0.00 | - | - | - | - | - | - | - | - | - | - |
| **Total Isopoda** | **0.77** | **0.04** | **3.33** | **1.35** | **0.04** | **-** | **-** | **-** | **-** | **-** | **-** | **-** | **-** | **-** | **-** |
| **Amphipoda** | | | | | | | | | | | | | | | |
| *Phtisica marina* | 0.00 | 0.01 | 1.67 | 0.03 | 0.00 | - | - | - | - | - | - | - | - | - | - |
| *Westwoodilla rectirostris* | 1.34 | 0.25 | 3.33 | 5.29 | 0.14 | - | - | - | - | - | - | - | - | - | - |
| *Ampelisca* sp. | 4.97 | 1.47 | 11.67 | 75.10 | 2.05 | - | - | - | - | - | - | - | - | - | - |
| *Monoculodes* sp. | 0.07 | 0.07 | 3.33 | 0.44 | 0.01 | - | - | - | - | - | - | - | - | - | - |
| Gammaridea | 0.41 | 0.07 | 6.67 | 3.17 | 0.09 | - | - | - | - | - | - | - | - | - | - |
| *Anchylomera blossevillei* | - | - | - | - | - | - | - | - | - | - | 5.28 | 11.52 | 25.00 | 420.05 | 3.92 |
| *Phronima atlantica* | - | - | - | - | - | 0.03 | 0.58 | 7.69 | 4.73 | 0.12 | - | - | - | - | - |
| Hyperiidae | - | - | - | - | - | - | - | - | - | - | 0.14 | 0.46 | 25.00 | 15.14 | 0.07 |

| | | | | | | | | | | | | | | | |
|---|---|---|---|---|---|---|---|---|---|---|---|---|---|---|---|
| Hyperiidea | - | - | - | - | - | 0.24 | 4.07 | 7.69 | 33.18 | 0.86 | - | - | - | - | - |
| **Total Amphipoda** | **6.79** | **1.86** | **26.67** | **84.03** | **2.30** | **0.28** | **4.65** | **15.38** | **37.91** | **0.99** | **5.43** | **11.98** | **50.00** | **435.19** | **3.99** |
| **Total Crustacea** | **69.80** | **72.13** | **148.33** | **2184.12** | **59.73** | **18.93** | **22.09** | **92.31** | **443.45** | **11.55** | **69.28** | **94.93** | **475.00** | **9780.32** | **85.57** |
| **OSTEICHTHYES** | | | | | | | | | | | | | | | |
| *E. encrasic*. larvae | - | - | - | - | - | 0 | 15.12 | 7.69 | 116.28 | 3.03 | - | - | - | - | - |
| Fish skeleton | 4.52 | 0.28 | 10.00 | 47.92 | 1.31 | 7.76 | 4.65 | 46.15 | 573.01 | 14.93 | 23.95 | 1.84 | 50.00 | 1289.53 | 51.28 |
| **Total Osteichthyes** | **4.52** | **0.28** | **10.00** | **47.92** | **1.31** | **7.76** | **19.77** | **53.85** | **689.29** | **17.95** | **23.95** | **1.84** | **50.00** | **1289.53** | **51.28** |
| **OTHERS** | | | | | | | | | | | | | | | |
| Unid.digestive pulp | 8.60 | 0.13 | 16.67 | 145.55 | 3.98 | - | - | - | - | - | 6.58 | 0.46 | 25.00 | 176.11 | 1.64 |
| Scales | 15.18 | 22.68 | 30.00 | 1136.01 | 31.07 | 1.32 | 53.49 | 38.46 | 2107.83 | 54.90 | - | - | - | - | - |
| *Anisakis* sp. | 0.03 | 0.18 | 10.00 | 2.14 | 0.06 | - | - | - | - | - | - | - | - | - | - |
| Trematoda parasites | 0.13 | 0.33 | 25.00 | 11.53 | 0.32 | 0.01 | 1.16 | 15.38 | 18.04 | 0.47 | - | - | - | - | - |
| Foraminifera | 0.97 | 3.71 | 23.33 | 109.25 | 2.99 | - | - | - | - | - | - | - | - | - | - |
| **Total Others** | **24.92** | **27.04** | **105.00** | **1404.47** | **38.41** | **1.32** | **54.65** | **53.85** | **2125.86** | **55.37** | **6.58** | **0.46** | **25.00** | **176.11** | **1.64** |

**Supplementary Table S11.** Output of SIMPER analysis conducted on the diet composition (%W) of *T. mediterraneus* within each Area (N= North Adriatic, C= Central Adriatic, S= South Adriatic). SIMPER analysis was conducted on the Bray Curtis resemblance matrix of transformed biomass data. Only comparisons between neighbouring sub-areas are shown. Cut-off for low contribution at 60%. Av.Abund = average abundance; Av. Sim = average similarity; Contrib %= percentage of variance explained by the explanatory variables; Cum. %= cumulative percentage of variance explained by the explanatory variables.

| | | **Group N<br>Average similarity: 2.71 %** | | |
|---|---|---|---|---|
| Species | Av.Abund | Av.Sim | Contrib% | Cum.% |
| *Acartia* spp. | 0.00 | 1.40 | 51.49 | 51.49 |
| *Siriella* sp. | 0.00 | 0.32 | 11.79 | 63.28 |
| | | **Group C<br>Average similarity: 3.31%** | | |
| Species | Av.Abund | Av.Sim | Contrib% | Cum.% |
| Fish skeleton | 0.01 | 2.08 | 62.90 | 62.90 |
| | | **Group S<br>Average similarity: 9.4%** | | |
| Species | Av.Abund | Av.Sim | Contrib% | Cum.% |
| Fish skeleton | 0.00 | 5.01 | 53.36 | 53.36 |
| *Calanus* spp. | 0.00 | 1.49 | 15.90 | 69.26 |
| | | **Groups N & C<br>Average dissimilarity = 97.93%** | | |
| | Group N | Group C | | |
| Species | Av.Abund | Av.Abund | Contrib% | Cum.% |
| Fish skeleton | 0.00 | 0.01 | 22.31 | 22.31 |
| *Alpheus glaber* | 0.00 | 0.01 | 12.66 | 34.97 |
| *Siriella* sp. | 0.00 | 0.00 | 10.46 | 45.43 |
| *Illex coindetii* | 0.00 | 0.03 | 7.68 | 53.11 |
| Mysidea | 0.00 | 0.00 | 7.63 | 60.74 |
| | | **Groups N & S<br>Average dissimilarity = 97.88%** | | |
| | Group N | Group S | | |
| Species | Av.Abund | Av.Abund | Contrib% | Cum.% |
| Fish skeleton | 0.00 | 0.00 | 22.21 | 22.21 |
| Squillidae larva | 0.00 | 0.00 | 18.30 | 40.51 |
| Zoea Caridea | 0.00 | 0.00 | 12.09 | 52.61 |
| *Eucheta* sp. | 0.00 | 0.00 | 8.23 | 60.83 |
| | | **Groups C & S<br>Average dissimilarity = 94.11%** | | |
| | Group C | Group S | | |
| Species | Av.Abund | Av.Abund | Contrib% | Cum.% |
| Fish skeleton | 0.01 | 0.00 | 22.71 | 22.71 |
| Squillidae larva | 0.00 | 0.00 | 14.29 | 37.00 |
| *Alpheus glaber* | 0.01 | 0.00 | 10.38 | 47.38 |
| Zoea Caridea | 0.00 | 0.00 | 9.65 | 57.03 |

| | | | | |
|---|---|---|---|---|
| *Illex coindetii* | 0.03 | 0 | 7.85 | 64.88 |

**Supplementary Table S12.** List of %W, %N, %F and %IRI values of stomach contents found in *Trachurus trachurus*, in North, Central and South Adriatic Sea.

| | North Adriatic | | | | | Central Adriatic | | | | | South Adriatic | | | | |
|---|---|---|---|---|---|---|---|---|---|---|---|---|---|---|---|
| | **W%** | **N%** | **F%** | **IRI** | **IRI%** | **W%** | **N%** | **F%** | **IRI** | **IRI%** | **W%** | **N%** | **F%** | **IRI** | **IRI%** |
| **MOLLUSCA** | | | | | | | | | | | | | | | |
| *Creseis acicula* | - | - | - | - | - | - | - | - | - | - | 0.05 | 3.36 | 12.50 | 42.58 | 0.89 |
| **Total Mollusca** | - | - | - | - | - | - | - | - | - | - | **0.05** | **3.36** | **12.50** | **42.58** | **0.89** |
| **CRUSTACEA** | | | | | | | | | | | | | | | |
| **Cladocera** | | | | | | | | | | | | | | | |
| Unid. Cladocera | - | - | - | - | - | - | - | - | - | - | 0.03 | 0.67 | 12.50 | 8.81 | 0.18 |
| **Total Cladocera** | - | - | - | - | - | - | - | - | - | - | **0.03** | **0.67** | **12.50** | **8.81** | **0.18** |
| **Copepoda** | | | | | | | | | | | | | | | |
| *Acartia* sp. | 96.11 | 99.31 | 25.00 | 4885.43 | 96.68 | - | - | - | - | - | - | - | - | - | - |
| *Calanus*-like | - | - | - | - | - | 0.14 | 3.28 | 9.09 | 31.05 | 0.22 | 0.40 | 16.11 | 25.00 | 412.76 | 8.66 |
| *Centropages* sp. | - | - | - | - | - | 0.05 | 1.46 | 4.55 | 6.85 | 0.05 | 0.08 | 4.70 | 37.50 | 179.32 | 3.76 |
| *Paraeuchaeta hebes* | - | - | - | - | - | 0.59 | 1.86 | 22.73 | 55.71 | 0.39 | 0.56 | 6.71 | 50.00 | 363.77 | 7.63 |
| *Euchaeta marina* | - | - | - | - | - | - | - | - | - | - | 0.64 | 7.38 | 25.00 | 200.51 | 4.21 |
| *Sapphirina* sp. | - | - | - | - | - | - | - | - | - | - | 0.02 | 0.67 | 12.50 | 8.60 | 0.18 |
| *Euterpina acutifrons* | - | - | - | - | - | - | - | - | - | - | 0.07 | 1.34 | 12.50 | 17.62 | 0.37 |
| Unid. Copepoda | - | - | - | - | - | 0.68 | 12.13 | 13.64 | 174.66 | 1.22 | - | - | - | - | - |
| **Total Copepoda** | **96.11** | **99.31** | **25.00** | **4885.43** | **96.68** | **1.46** | **18.72** | **50.00** | **268.28** | **1.87** | **1.77** | **36.91** | **162.50** | **1182.58** | **24.81** |
| **Ostracoda** | | | | | | | | | | | | | | | |
| Unid. Ostracoda | - | - | - | - | - | - | - | - | - | - | 0.10 | 0.67 | 12.50 | 9.65 | 0.20 |
| **Total Ostracoda** | - | - | - | - | - | - | - | - | - | - | **0.10** | **0.67** | **12.50** | **9.65** | **0.20** |
| **Euphausiacea** | | | | | | | | | | | | | | | |
| *Nematoscelis sp.* | - | - | - | - | - | - | - | - | - | - | 0.97 | 2.01 | 12.50 | 37.34 | 0.78 |
| *Nyctiphanes couchii* | - | - | - | - | - | 94.28 | 76.47 | 81.82 | ####### | 97.62 | 18.84 | 10.74 | 37.50 | 1109.04 | 23.27 |
| Euphausiidae larvae | - | - | - | - | - | - | - | - | - | - | 0.30 | 6.71 | 25.00 | 175.34 | 3.68 |
| Euphausiidae | - | - | - | - | - | 2.54 | 3.35 | 4.55 | 26.78 | 0.19 | 6.88 | 3.36 | 12.50 | 127.99 | 2.69 |
| **Total Euphausiacea** | - | - | - | - | - | **96.83** | **79.83** | **86.36** | **#######** | **97.81** | **27.00** | **22.82** | **87.50** | **1449.71** | **30.42** |
| **Decapoda** | | | | | | | | | | | | | | | |
| Megalopa Brachyura | - | - | - | - | - | 0.00 | 0.04 | 4.55 | - | - | - | - | - | - | - |
| Zoea Penaeidae | - | - | - | - | - | - | - | - | - | - | 0.27 | 0.67 | 12.50 | 11.75 | 0.25 |

| | | | | | | | | | | | | | | | |
|---|---|---|---|---|---|---|---|---|---|---|---|---|---|---|---|
| **Total Decapoda** | - | - | - | - | - | - | - | - | - | - | **0.27** | **0.67** | **12.50** | **11.75** | **0.25** |
| **Amphipoda** | | | | | | | | | | | | | | | |
| *Themisto gaudichaudii* | 1.11 | 0.11 | 25.00 | 30.68 | 0.61 | - | - | - | - | - | - | - | - | - | - |
| *Themisto abyssorum* | - | - | - | - | - | - | - | - | - | - | 0.82 | 2.01 | 12.50 | 35.45 | 0.74 |
| Hyperiidae | - | - | - | - | - | 0.33 | 1.13 | 18.18 | 26.59 | 0.19 | 7.35 | 26.17 | 25.00 | 838.19 | 17.59 |
| **Total Amphipoda** | **1.11** | **0.11** | **25.00** | **30.68** | **0.61** | **0.33** | **1.13** | **18.18** | **26.59** | **0.19** | **8.18** | **28.19** | **37.50** | **873.64** | **18.33** |
| **Total Crustacea** | **97.22** | **99.43** | **50.00** | **4916.11** | **97.29** | **98.62** | **99.67** | **154.55** | ####### | **99.87** | **37.35** | **89.93** | **325.00** | **3536.14** | **74.19** |
| **OSTEICHTHYES** | | | | | | | | | | | | | | | |
| *E. encrasic.* larvae | - | - | - | - | - | - | - | - | - | - | 36.01 | 3.36 | 12.50 | 492.08 | 10.32 |
| *Serranus* sp. larvae | - | - | - | - | - | - | - | - | - | - | 2.08 | 0.67 | 12.50 | 34.41 | 0.72 |
| **Total Osteichthyes** | - | - | - | - | - | - | - | - | - | - | **38.09** | **4.03** | **25.00** | **526.49** | **11.05** |
| **OTHERS** | | | | | | | | | | | | | | | |
| Unid.digestive pulp | - | - | - | - | - | 1.14 | 0.11 | 13.64 | 17.08 | 0.12 | 24.36 | 1.34 | 25.00 | 642.55 | 13.48 |
| Scales | 1.11 | 0.11 | 25.00 | 30.68 | 0.61 | 0.23 | 0.18 | 4.55 | 1.88 | 0.01 | - | - | - | - | - |
| *Anisakis* sp. | - | - | - | - | - | - | - | - | - | - | 0.15 | 1.34 | 12.50 | 18.67 | 0.39 |
| Trematoda parasites | 1.67 | 0.46 | 50.00 | 106.41 | 2.11 | - | - | - | - | - | - | - | - | - | - |
| **Total Others** | **2.78** | **0.57** | **75.00** | **137.10** | **2.71** | **1.37** | **0.29** | **18.18** | **18.96** | **0.13** | **24.51** | **2.68** | **37.50** | **661.21** | **13.87** |

**Supplementary Table S13.** Output of SIMPER analysis conducted on the diet composition (%W) of *T. trachurus* within each Area (N= North Adriatic, C= Central Adriatic, S= South Adriatic). SIMPER analysis was conducted on the Bray Curtis resemblance matrix of transformed biomass data. Only comparisons between neighbouring sub-areas are shown. Cut-off for low contribution at 60%. Av.Abund = average abundance; Av. Sim = average similarity; Contrib %= percentage of variance explained by the explanatory variables; Cum. %= cumulative percentage of variance explained by the explanatory variables.

| | | | | |
|---|---|---|---|---|
| | | **Group N**<br>**Average similarity: -%** | | |
| | | | | |
| | | **Group C**<br>**Average similarity: 37.86%** | | |
| Species | Av.Abund | Av.Sim | Contrib% | Cum.% |
| *Nyctiphanes couchii* | 0.06 | 37.77 | 99.78 | 99.78 |
| | | **Group S**<br>**Average similarity: 5.38%** | | |
| Species | Av.Abund | Av.Sim | Contrib% | Cum.% |
| *Nyctiphanes couchii* | 0.01 | 2.73 | 50.71 | 50.71 |
| *Calanus* spp. | 0.00 | 0.95 | 17.62 | 68.33 |
| | | **Groups N & C**<br>**Average dissimilarity = 100%** | | |
| | Group N | Group C | | |
| Species | Av.Abund | Av.Abund | Contrib% | Cum.% |
| *Nyctiphanes couchii* | 0.00 | 0.06 | 77.30 | 77.30 |
| | | **Groups N & S**<br>**Average dissimilarity = 100.00%** | | |
| | Group N | Group S | | |
| Species | Av.Abund | Av.Abund | Contrib% | Cum.% |
| *Nyctiphanes couchii* | 0 | 0.01 | 19.12 | 19.12 |
| Fam. Hyperidae | 0 | 0 | 15.33 | 34.45 |
| *Engraulis encrasicolus* larvae | 0 | 0.01 | 10.42 | 44.87 |
| Euphasids larvae | 0 | 0 | 8.66 | 53.53 |
| Copepoda | 0 | 0 | 8.57 | 62.11 |
| | | **Groups C & S**<br>**Average dissimilarity = 90.71%** | | |
| | Group C | Group S | | |
| Species | Av.Abund | Av.Abund | Contrib% | Cum.% |
| *Nyctiphanes couchii* | 0.06 | 0.01 | 67.98 | 67.98 |

**Supplementary Table S14.** Output of SIMPER analysis conducted on the diet composition (%W) of all the four "ancillary" pelagic species. SIMPER analysis was conducted on the Bray Curtis resemblance matrix of transformed biomass data. Cut-off for low contribution at 60%. Av.Abund = average abundanceContrib %= percentage of variance explained by the explanatory variables; Cum. %= cumulative percentage of variance explained by the explanatory variables.

| | | | | |
|---|---|---|---|---|
| | | **Groups *Scomber colias vs. Scomber scomber*** | | |
| | | **Average dissimilarity = 97.99 %** | | |
| | *S. colias* | *S. scombrus* | | |
| Species | Av.Abund | Av.Abund | Contrib% | Cum.% |
| Salpidae | 0.05 | 0.00 | 42.77 | 42.77 |
| Fish skeleton | 0.01 | 0.00 | 15.96 | 58.74 |
| *E.encrasicolus* larvae | 0.03 | 0.00 | 5.70 | 64.43 |
| | | **Groups *Scomber colias vs. Trachurus mediterraneus*** | | |
| | | **Average dissimilarity = 99.08 %** | | |
| | *S. colias* | *T. mediterraneus* | | |
| Species | Av.Abund | Av.Abund | Contrib% | Cum.% |
| Salpidae | 0.05 | 0.00 | 39.95 | 39.95 |
| Fish skeleton | 0.01 | 0.00 | 14.50 | 54.45 |
| *E.encrasicolus* larvae | 0.03 | 0.00 | 6.41 | 60.86 |
| | | **Groups *Scomber scombrus vs. Trachurus mediterraneus*** | | |
| | | **Average dissimilarity = 93.77 %** | | |
| | *S. scombrus* | *T. mediterraneus* | | |
| Species | Av.Abund | Av.Abund | Contrib% | Cum.% |
| Fish skeleton | 0.00 | 0.00 | 17.26 | 17.26 |
| *Acartia* sp. | 0.00 | 0.00 | 11.14 | 28.40 |
| *Pyrosoma* sp. | 0.00 | 0.00 | 8.30 | 36.70 |
| *Siriella* sp. | 0.00 | 0.00 | 7.58 | 44.29 |
| Clupeiforme | 0.02 | 0.00 | 6.78 | 51.07 |
| Sepiolidae | 0.00 | 0.00 | 4.70 | 55.77 |
| *Alpheus glaber* | 0.00 | 0.00 | 4.18 | 59.94 |
| *Philoceras bispinosus* | 0.00 | 0.00 | 2.97 | 62.92 |
| | | **Groups *Scomber colias vs. Trachurus trachurus*** | | |
| | | **Average dissimilarity = 98.63 %** | | |
| | *S. colias* | *T. trachurus* | | |
| Species | Av.Abund | Av.Abund | Contrib% | Cum.% |
| Salpidae | 0.05 | 0.00 | 31.11 | 31.11 |
| *Nyctiphanes couchii* | 0.02 | 0.04 | 29.05 | 60.16 |
| | | **Groups *Scomber scombrus vs. Trachurus trachurus*** | | |
| | | **Average dissimilarity = 99.37 %** | | |
| | *S. scombrus* | *T. trachurus* | | |
| Species | Av.Abund | Av.Abund | Contrib% | Cum.% |
| *Nyctiphanes couchii* | 0.00 | 0.04 | 41.25 | 41.25 |
| Clupeiforme | 0.02 | 0.00 | 5.80 | 55.03 |
| Copepoda | 0.00 | 0.00 | 5.43 | 60.46 |
| | | **Groups *Trachurus mediterraneus vs. Trachurus trachurus*** | | |
| | | **Average dissimilarity = 98.54 %** | | |
| | *T. mediterraneus* | *T. trachurus* | | |
| Species | Av.Abund | Av.Abund | Contrib% | Cum.% |

| | | | | |
|---|---|---|---|---|
| *Nyctiphanes couchii* | 0.00 | 0.04 | 46.51 | 46.51 |
| *Acartia* sp. | 0.00 | 0.00 | 5.76 | 52.27 |
| Fish skeleton | 0.00 | 0.00 | 4.86 | 57.12 |
| *Siriella* sp. | 0.00 | 0.00 | 4.04 | 61.17 |

**Supplementary Table S15.** Output of PERMDISP multivariate dispersion test conducted on values of the diet composition (%W) of the four "ancillary" pelagic species. t=statistic t for pairwise comparisons; SE=standard error; P(perm)=probability level; ***=p ≤ 0.001; n.s. = not significant.

DEVIATIONS FROM CENTROID

F: 11.091

P(perm): ***

| **PAIRWISE COMPARISONS** | | | |
|---|---|---|---|
| **Groups** | **t** | **P(perm)** | |
| *S. colias vs. S. scombrus* | 1.32 | n.s. | |
| *S. colias vs. T. mediterraneus* | 5.88 | *** | |
| *S. colias vs. T. mediterraneus* | 0.34 | n.s. | |
| *S. scombrus vs. T. mediterraneus* | 6.19 | *** | |
| *S. scombrus vs. T. trachurus* | 0.67 | n.s. | |
| *T. mediterraneus vs. T. trachurus* | 4.27 | *** | |
| **MEANS AND STANDARD ERRORS** | | | |
| **Group** | **Size** | **Average** | **SE** |
| *S. colias* | 62 | 58.78 | 1.33 |
| *S. scombrus* | 16 | 54.63 | 3.43 |
| *T. mediterraneus* | 74 | 66.75 | 0.55 |
| *T. trachurus* | 34 | 57.83 | 2.86 |

**Supplementary Table S16.** Mean values of $\delta^{13}C_{corrected}$ (‰) and $\delta^{15}N$ (‰) measured in the specimens belonging to the four "ancillary" pelagic species caught in the North, Central, and South Adriatic Sea. S.D. =standard deviation.

| **Species** | **Area** | **$\delta^{13}C_{corrected}$** | | **$\delta^{15}N$** | | **C/N** | |
|---|---|---|---|---|---|---|---|
| | | mean | S.D. | mean | S.D. | mean | S.D. |
| *Scomber colias* | North | -18.4 | 0.4 | 8.7 | 1.6 | 2.7 | 0.6 |
| | Central | -18.6 | 0.1 | 10.3 | 0.6 | 2.8 | 1.2 |
| | South | -18.2 | 0.4 | 8.2 | 0.9 | 2.7 | 0.9 |
| *Scomber scombrus* | North | -19.3 | 0.8 | 11.1 | 1.2 | 2.8 | 0.1 |
| | Central | -18.6 | 0.4 | 11.1 | 0.2 | 2.9 | 0.2 |
| *Trachurus mediterraneus* | North | -18.4 | 0.6 | 10.9 | 1.3 | 2.8 | 0.1 |
| | Central | -18.4 | 1.1 | 10.5 | 1.5 | 2.9 | 0.1 |
| | South | -18.9 | 0.2 | 7.6 | 0.5 | 2.8 | 0.1 |
| *Trachurus trachurus* | North | -19.3 | 0.3 | 10.5 | 0.5 | 2.8 | 0.1 |
| | Central | -19.1 | 0.2 | 9.3 | 1.5 | 2.9 | 0.1 |
| | South | -19.4 | 0.4 | 8.4 | 1.6 | 2.8 | 0.2 |

**Supplementary Table S17.** Results of univariate PERMANOVA main and pairwise tests carried out on the $\delta^{13}C$, $\delta^{15}N$ and C:N values of the four "ancillary" pelagic species. Pairwise comparisons are conducted on the term "Species". df=degrees of freedom; MS= mean square; Pseudo-F= statistic F; t=statistic t for pairwise comparisons; P(MC)= probability level after Monte Carlo test; *=p < 0.05; **=p < 0.01; ***=p < 0.001; n.s. = not significant.

| PERMANOVA main test on $\delta^{13}C$ | | | | | |
|---|---|---|---|---|---|
| **Species** | **Source** | **df** | **MS** | **Pseudo-F** | **P(MC)** |
| All species | Species | 3 | 2.94 | 5.46 | * |
| | Area(Species) | 7 | 0.55 | 1.74 | n.s. |
| | Residuals | 80 | 0.32 | | |
| | Total | 90 | | | |

| PAIRWISE comparison among species on values of $\delta^{13}C$ | | |
|---|---|---|
| **Groups** | **t** | **P(MC)** |
| *S. colias vs. S. scombrus* | 1.81 | n.s. |
| *S. colias vs. T. mediterraneus* | 0.86 | n.s. |
| *S. colias vs. T. mediterraneus* | 6.18 | ** |
| *S. scombrus vs. T. mediterraneus* | 1.13 | n.s. |
| *S. scombrus vs. T. trachurus* | 1.11 | n.s. |
| *T. mediterraneus vs. T. trachurus* | 3.55 | * |

| PERMANOVA main test on $\delta^{15}N$ | | | | | |
|---|---|---|---|---|---|
| **Species** | **Source** | **df** | **MS** | **Pseudo-F** | **P(MC)** |
| All species | Species | 3 | 12.24 | 1.17 | n.s. |
| | Area(Species) | 7 | 10.96 | 7.53 | *** |
| | Residuals | 80 | 1.45 | | |
| | Total | 90 | | | |

| PERMANOVA main test on $\delta^{13}C$ and $\delta^{15}N$ | | | | | |
|---|---|---|---|---|---|
| **Species** | **Source** | **df** | **MS** | **Pseudo-F** | **P(MC)** |
| All species | Species | 3 | 71.77 | 1.52 | n.s. |
| | Area(Species) | 7 | 49.22 | 6.07 | *** |
| | Residuals | 80 | 8.11 | | |
| | Total | 90 | | | |

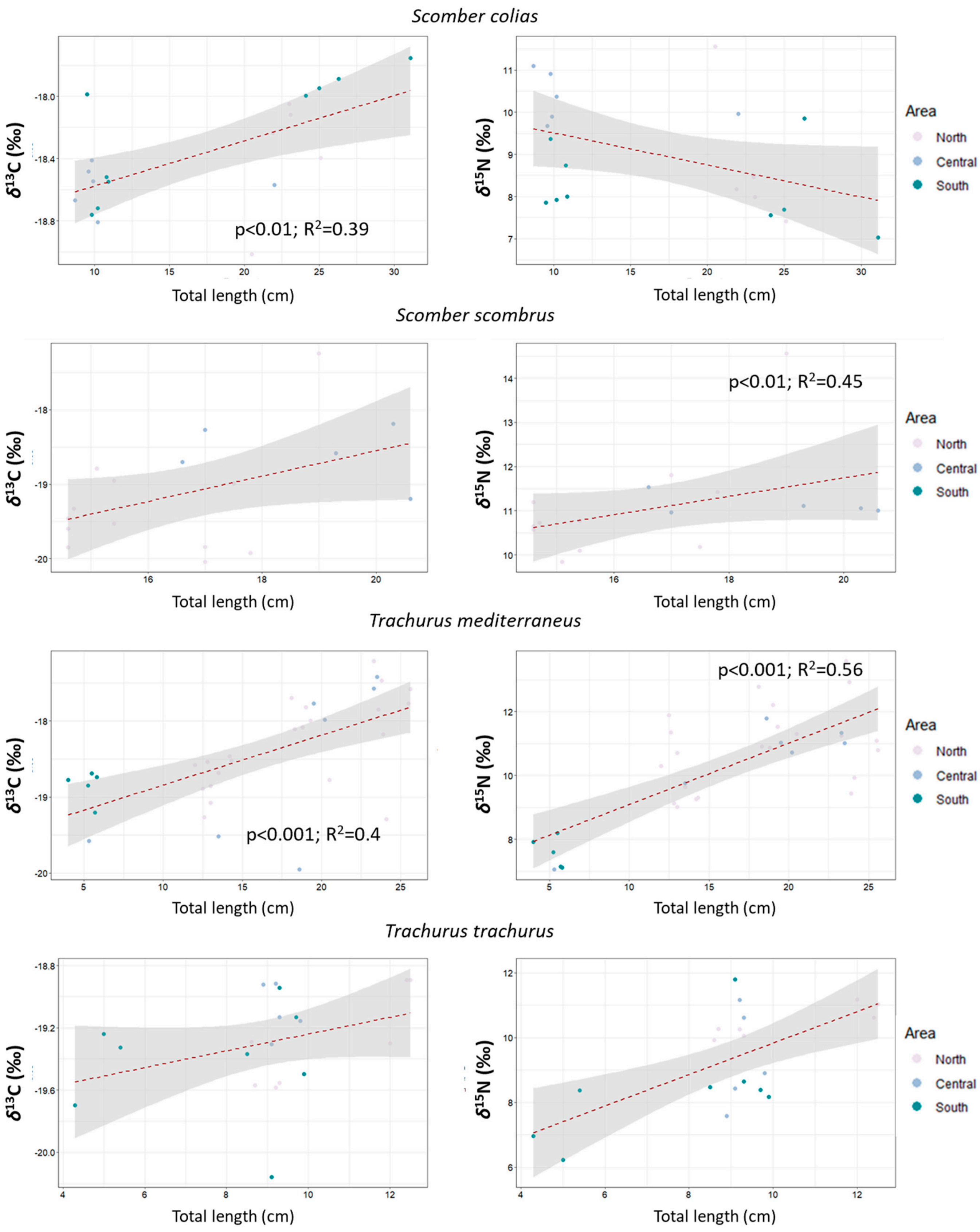


**Supplementary Figure S1.** Correlation of total length (expressed in cm) with values of $\delta^{13}C$ (figures on the left) and $\delta^{15}N$ (figures on the right) measured in the individuals of the four "ancillary" pelagic species caught in the North, Central and South Adriatic Sea.

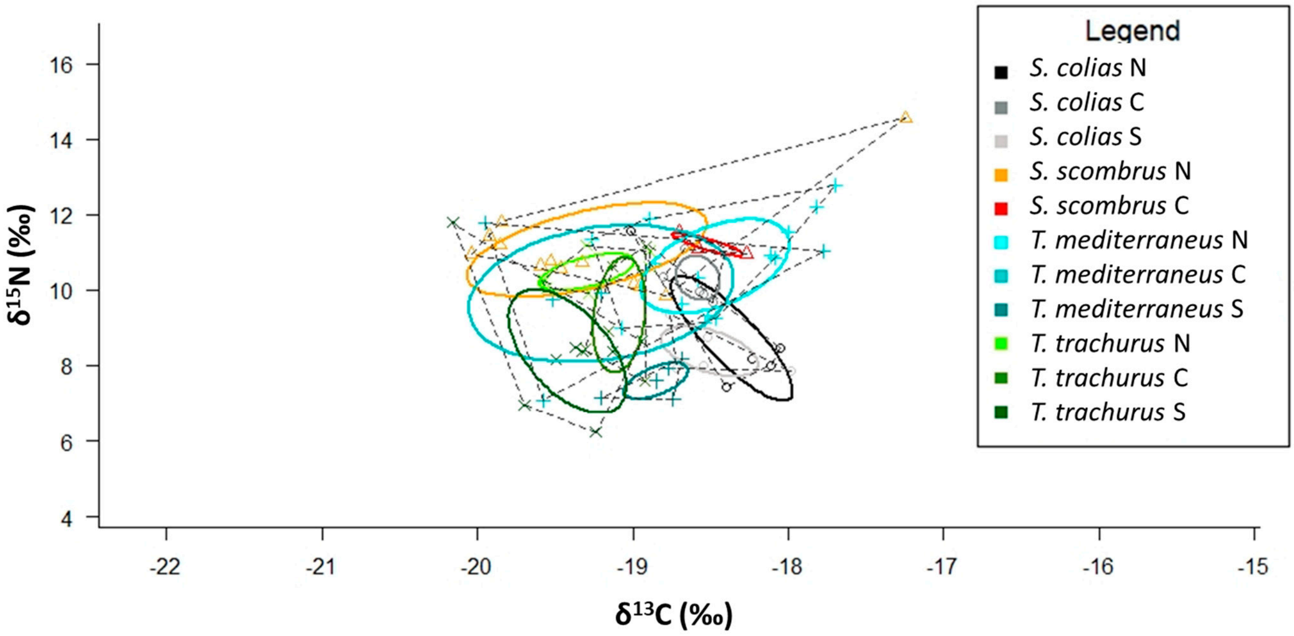


**Supplementary Figure S2.** $\delta^{13}C$ - $\delta^{15}N$ scatterplot with standard ellipses corrected for small sample size population ($SEA_C$, ‰$^2$) overlaid for the specimens of the four "ancillary" pelagic species collected in the North (N), Central (C) and South (S) Adriatic Sea.
